# Myrcene—What Are the Potential Health Benefits of This Flavouring and Aroma Agent?

**DOI:** 10.3389/fnut.2021.699666

**Published:** 2021-07-19

**Authors:** Shelini Surendran, Fatimah Qassadi, Geyan Surendran, Dash Lilley, Michael Heinrich

**Affiliations:** ^1^Faculty of Health and Medical Sciences, University of Surrey, Guildford, United Kingdom; ^2^Pharmacognosy and Phytotherapy, University College London (UCL) School of Pharmacy, London, United Kingdom; ^3^Graduate Institute of Integrated Medicine, College of Chinese Medicine, Chinese Medicine Research Center, China Medical University, Taichung, Taiwan; ^4^Beyond Alcohol Ltd., London, United Kingdom

**Keywords:** myrcene, hop, toxicology, phytochemistry, biological activities, plant biotechnology, non-alcoholic beer

## Abstract

Myrcene (β-myrcene) is an abundant monoterpene which occurs as a major constituent in many plant species, including hops and cannabis. It is a popular flavouring and aroma agent (food additive) used in the manufacture of food and beverages. This review aims to report on the occurrence, biological and toxicological profile of β-myrcene. The main reported biological properties of β-myrcene—anxiolytic, antioxidant, anti-ageing, anti-inflammatory, analgesic properties—are discussed, with the mechanisms of activity. Here we also discuss recent data regarding the safety of β-myrcene. Overall, β-myrcene has shown promising health benefits in many animal studies. However, studies conducted in humans is lacking. In the future, there is potential for the formulation and production of non-alcoholic beers, functional foods and drinks, and cannabis extracts (low in THC) rich in β-myrcene.

## Introduction

Myrcene (7-methyl-3-methylene-1,6-octadiene) is a popular food additive used as a flavouring agent in the manufacture of food and beverages (1). It is further used in consumer products, such as cosmetics, soaps, and detergents. In addition to its use in a variety of consumable products, β-Myrcene is used as a starting material for commercially important scents and flavours such as menthol, nerol, geraniol, and linalool (2). β-Myrcene has a high production volume of 58,076 kg for Europe and 1,188 kg for the USA (3).

β-Myrcene is a pleasant-smelling, olefinic, acyclic unsubstituted monoterpene which occurs naturally in a large number of plant species (4–6), especially in the essential oils of plants such as hops, cannabis, lemongrass, verbena and bay (4, 7), as well as in citrus fruits and citrus juices (8).

In brewing, β-myrcene is one of the most potent aromatic flavour components of hop essential oils and in all analysed hop varieties is considered the most odour-active volatile (9, 10). Myrcene largely determines the “green hop aroma” in beer and is a primary substance in dry hopped beers (11), with a “herbaceous, resinous, green, balsamic, fresh hop” like odour (12, 13). It is also the major constituent of hop essential oil and can constitute as much as 70% of the essential oil by volume (14).

In addition to the flavour of hops, β-myrcene contributes significantly to cannabis aromas, and may function analogously to the endocannabinoid system. β-Myrcene characteristically gives cannabis strains a mildly sweet flavour profile and provides scent notes that are spicy, earthy and musky (15). Cannabis strains which contain high concentrations of myrcene (>0.5% myrcene), are likely to induce sedative qualities (“couch-lock effect”), which are classically attributed to *Cannabis indica* Lam (a synonym of *C. sativa* L.) strains (16). On the other hand, strains low in β-myrcene (<0.5%) are likely to induce a more energic “high” (17). β-Myrcene may also have a role in assisting cannabinoids to be absorbed across the blood-brain barrier, increasing transport into the brain and enhancing psychoactive responses; however, there is limited robust data supporting this claim (18).

β-Myrcene reported biological activities include analgesic (19), sedative (20), antidiabetic (21), antioxidant (22), anti-inflammatory (23), antibacterial (24), and anticancer effects (25).

Despite the therapeutic benefits observed, β-myrcene has come under scientific scrutiny due to an alleged risk as a potential human carcinogen. The uncertainty of the safety of myrcene, stems from studies conducted by the National Toxicology Program, USA (NTP) which has showed an increased incidence of kidney and liver neoplasms in rodents (26). In 2018, the FDA took regulatory action to no longer permit the use of β-myrcene, as a food additive based on legal action taken against the FDA under the Delaney Clause (A federal health statute which prohibits FDA approval of any food additive which caused cancer in humans or animals). Importantly, the FDA confirmed that there was no safety concern for β-myrcene to public health under the conditions of its intended use. Several other regulatory and scientific expert bodies have since argued that β-myrcene is safe under conditions of intended use as a flavouring substance (27) and it must be noted that countless permitted food products continue to naturally contain significant levels of β- myrcene.

The wide application of β-myrcene in industry and for domestic use coupled with safety concerns, has raised the interest to critically review its biochemical and pharmacological properties. Thus, the aim of this review is to explore and assess some of the important biological activities of β-myrcene and to assess its suitability for commercial use in food industry and phytotherapy.

## Approach and Methods

This review paper collected the literature published on the phytochemistry, pharmacodynamics, pharmacology, pharmacokinetics, health benefits and occurrence of myrcene in different plant species and toxicity. Relevant information on myrcene was gathered from worldwide accepted scientific search engines and databases, including Web of Science, PubMed, Elsevier, Wiley Online Library, ResearchGate and Google Scholar. The key words used for the searches were “myrcene,” combined with “monoterpene,” “phytochemistry,” “pharmacokinetics,” “pharmacology”; specialist pharmacological terms included,” “anxiolytic,” “sedative” “antioxidant” “skin” “anti-inflammatory” “pain,” “analgesic,” and “toxicology.” Most of the cited information in this article were from peer-reviewed journals directly published in English. No time period limitation was considered. In addition, reference lists of identified publications were hand searched to identify other studies potentially eligible for inclusion. Species names were cross-checked in MPNS (https://mpns.science.kew.org/).

### Physical and Chemical Properties of β-Myrcene

Monoterpenes are a class of terpenes; consisting of two isoprene units (5 carbon bases each) (28). Myrcene (C_10_H_16_, molecular weight 136.23 g/mol) (29) is classified as an acyclic monoterpene, with properties listed in Table 1.

**Table 1:** Chemical and physical properties of β-myrcene.

**Parameter**	**Value/Description**	**References**
Appearance	Colourless clear liquid or yellow oily liquid	(2, 29)
Odour description	Resinous, herbaceous, balsamic, and geranium-like	(30, 31)
Melting point (°C)	<-10	(32)
Boiling point at 1,013 hPa (°C)	167.1	(33)
Specific gravity at 25°C [g cm^−3^]	0.7847	(2)
Solubility	Practically insoluble in water. Soluble in alcohol, chloroform, ether, and glacial acetic acid	(34)
Stability	Polymerizes spontaneously at room temperature, whether air is excluded or not	(2)
Refractive index at 25°C	1.4660–1.4710	(2)
Flash point (°C)	44	(35)

**Figure 1:**
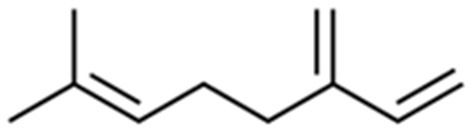
β-myrcene structure.

Myrcene exists in two isomeric forms, namely β-myrcene (7-methyl-3-methylene-1,6-octadiene) and α-myrcene (2-methyl-6-methylene-1,7-octadiene). The most common is the naturally occurring isomer β-myrcene, which contains an isopropylidene group and is often denoted as “myrcene” in the literature. The other is α-myrcene, which exists in the isopropenyl form (2). In β-myrcene there are three carbon-carbon double bonds (two of them being conjugated) and a gem-dimethyl terminal (Figure 1) (36).

### Biosynthesis Production of β-Myrcene

Monoterpenes are produced in plants by the stereospecific condensation of two isomeric five-carbon units: isopentenyl diphosphate (IDP) and dimethylallyl diphosphate (DMADP) (37). Geranyl diphosphate (GPP) is thus formed via condensation of IPP with DMADP, by GPP synthase (GPPS) (38, 39). Geranyl diphosphate (GPP) then undergoes hydrolysis catalysed by a prenolpyrophosphatase to form geraniol. Consequently, β-myrcene is produced by the dehydration and isomerization of geraniol (37).

### Industrial Synthesis of Myrcene

β-Pinene is an important starting material for the synthesis of β- myrcene. Up until the 1950s, β-pinene was extracted from pine or spruce resins by tapping trees Nowadays, β-pinene can be isolated from the waste streams of paper mills, by distillation and desulphurisation from crude sulphate turpentine (CST). The proportion of β-pinene in CST, depends largely on the age, season, geographical location, and variety of the tree (40).

The most common method to produce myrcene industrially is through the pyrolysis of β-pinene (2). In the future, engineered microbial platforms may provide alternative means for sustainable and environmentally friendly large-scale production of myrcene (41–43).

### Analysis, Extraction, and Quantification of β-Myrcene

β-Myrcene is considered an important intermediate compound which can be derivatized to produce numerous end products, such as, citronellol, citronellal, geraniol, nerol and linalool (2). Several methods of determining β-myrcene content have been published and developed to achieve more simple, rapid and efficient quantitative analytical methods (Table 2) (55, 58). Naturally, β-myrcene exists as complex mixtures with other monoterpenes. Thus, it would be difficult to isolate β-myrcene in large quantities from these complex mixtures. Therefore, there is a need for an efficient and economical method for separating β-myrcene from other terpenes. Hydro-distillation and solvent extraction were used extensively followed by Gas Chromatography (GC) coupled with Mass Spectrometry (MS) or Flame ionisation detector (FID) for quantification (58, 59). The drawbacks of these techniques were mainly the tendency of losing essential volatile compounds (including β-myrcene) during the process of solvent evaporation leading to decrease the yield and altering its odour characteristics (59, 60). Also, these methods require a lengthy process from 3 to 4 h of distillation allowing possibility of changing the chemical composition of analyte or risk of indefinable loss (51, 61).

**Table 2:** Extraction, detection and analysis of β-myrcene.

**Plant species**	**Family**	**Part (s) used** **for extraction/detection**	**Extraction method**	**Assay procedure**	**References**
*Cannabis sativa* L.	Cannabaceae	Female flower tops (“Cannabis Flos”)	Solvent extraction	Gas chromatography/flame ionisation detection- Nuclear magnetic resonance (GC-FID-MR)	(44)
*Cannabis sativa* L.	Cannabaceae	Flowers	Exhaustive solvent extraction	Gas chromatography–mass spectrometry (GC-MS)	(45)
*Citrus aurantium* L.	Rutaceae	Flower	Hydrodistillation and ultrasonic-assisted headspace solid phase microextraction	Gas Chromatography–Mass Spectrometry (GC-MS)	(46)
*Citrus maxima* (Burm.) Merr.	Rutaceae	Peels	Dynamic headspace collection	Thermal desorption system–gas chromatography–mass spectrometry (TD-GC/MS)	(47)
*Citrus × aurantium* L.	Rutaceae	Fruit	Solid phase microextraction	Gas-chromatography–olfactometry (GC-O)	(48)
*Citrus × aurantium* L.	Rutaceae	Peel, leaf	Hydrodistillation	Gas Chromatography–Mass Spectrometry-Nuclear Magnetic Resonance (GC-MS- NMR)	(49)
*Humulus lupulus* L.	Cannabaceae	Cones and leaves of hop	Supercritical carbon dioxide extraction	High-performance liquid chromatography- Gas Chromatography (HPLC/GC)	(50)
*Humulus lupulus* L.	Cannabaceae	Pellets and cones	HS trap	Gas Chromatography–Mass Spectrometry (GC-MS)	(51)
*Humulus lupulus* L.	Cannabaceae	Pellets	Stir bar-sorptive extraction	Gas Chromatography–Mass Spectrometry (GC-MS)	(52)
*Humulus lupulus* L.	Cannabaceae	Hop-essential oil	Solid phase microextraction	Gas Chromatography-quadrupole mass spectrometry (GC-qMS)	(53)
*Humulus lupulus* L.	Cannabaceae	Cones	HS-SPME and HD	Gas Chromatography (GC)	(54)
*Pistacia lentiscus* var. *Chia*	Anacardiaceae	The gum essential oil	Steam distillation	Fourier transform Raman spectroscopy (NIR FT-Raman)	(55)
*Pistacia lentiscus* var. *chia*	Anacardiaceae	The gum essential oil	Headspace Solid Phase Microextraction	Gas Chromatography–Mass Spectrometry (GC-MS)	(56)
*Spondias mombin* L.	Anacardiaceae	Fruit	Solid phase microextraction- simultaneous distillation and extraction	Gas Chromatography- Mass Spectrometry (GC-MS)	(57)

In contrast, extraction efficiency and flexibility of supercritical fluid CO_2_ extraction are much better than the earlier methods, since there is a greater control in temperature and pressure parameters (50). The yield of extracts obtained by supercritical CO_2_ extraction is highly dependent on the temperature and pressure applied during the extraction process. Higher recovery percentage of β-myrcene was obtained at lower temperatures and pressures of supercritical fluid (62). Also, at 40°C and 20.0 MPa, β- myrcene, L-caryophyllene and α-humulene were detected at higher concentrations allowing for their importance in beverage industry as flavouring and aroma additives (63). The demand for using environmentally clean extraction techniques and the need for faster, more powerful, and cheaper analytical procedures is therefore driving the industrial production into methods like supercritical fluid CO_2_ extraction.

All these techniques had noticeable disadvantages such as excessive use of solvents, longer operation time and/ or large volumes of sample (60, 62). Also, they require the use of highly sophisticated, uneconomical devices with a limited lifetime (53). Furthermore, these techniques are quite complex in operation and are labour intensive, thus, they are not fully efficient when a routine analysis of a large number of samples is needed.

Solid phase microextraction (SPME) has emerged as an alternative to traditional techniques (53). It has been successfully employed to determine the volatile composition of hop and also in different food products (54, 64). This technique has its own characteristics, such as efficient extraction procedures, short analysis of time, economical with low production cost, and high selectivity and sensitivity when coupled with suitable detection modes. Moreover, by using SPME, all the steps of extraction technique can be introduced within a single process without any interruption, resulting in a high sample processing (53).

SPME can also be used with (GC) and (MS) analysis, to provide a full metabolomic profile of the intended plant (53, 65, 66). Hops of the *Saaz* variety have been studied by Gonçalves et al. using SPME, allowing for profiling the terpenoid metabolomic pattern found in the essential oil. β-Myrcene was dominated by 53.0% of the total volatile fraction at 40°C. The disadvantages of SPME are that the extraction conditions, age of fibre, and matrix could affect the amount of sample absorbed on the fibre. Controlling these factors can produce a highly efficient and reproducible method for extraction and analysis of β-myrcene. Fourier transform Raman Spectroscopy has also been used to determine the percentage of β-myrcene in mastic gum oil based on band intensity measurements (55). The method is extremely rapid, simple and non-destructive for the sample.

There are still some limitations in obtaining a pure extract of β-myrcene allowing for accurate analysis and quantification. Thus, proper selection of extraction techniques and pre- determined sample parameters are necessary for efficient analysis of β-myrcene.

### Pharmacokinetics of β-Myrcene

Most previously published data on the absorption, distribution, metabolism and excretion of β-myrcene have been conducted in experimental animals, such as, rabbits and rats (26). In a pharmacokinetic study, blood levels as high as 14.1 ± 3.0 μg/mL β-myrcene (peak value) were detected 60 min after oral administration of 1.0 g/kg bw β-myrcene to female rats (67). β-Myrcene showed a pattern of elimination mostly by urine with an elimination half-life of 285 min, however no studies examined the possibility of biliary excretion (3, 67). β-Myrcene was mainly distributed in adipose tissue and in several major organs, including the liver, brain, kidneys, and gonads. β-Myrcene is bioavailable in human plasma within 30 min after consumption of a single dose. The high degree of bioavailability in plasma is a crucial step towards its useful usage in the food and beverage industry. Furthermore, β-myrcene reaches the plasma unaltered, with a peak concentration between 2 and 4 h (68). This could partially explain its health benefits and applications on human health. More research is needed to characterise the kinetics of β-myrcene in human metabolism.

Urinary excretion of conjugates of two diols (10-hydroxylinalool and 7-methyl-3-methylene-oct-6-ene-1,2-diol), and two hydroxy acids (10-carboxylinalool and 2-hydroxy-7-methyl-3-methylene-oct-6-enoic acid) was observed in male rabbits administered β-myrcene by gavage (69, 70). Diols were formed due to an oxidation reaction occurred at the 3,10-double bond through a 3,10-epoxide intermediate. The metabolites were isolated by using rat-liver microsomal cytochrome P450 enzymes and confirmed by undergoing enzymatic degradation by β-Glucuronidase/Arylsulfatase.

In rats, several metabolites were isolated from the urine after oral administration of β-myrcene, such as, 10-hydroxylinalool, 1-hydroxymethyl-4-isopropenyl cyclohexanol, 7-methyl-3-methylene-oct-6-ene-1,2-diol, 10-carboxylinalool, 2-hydroxy-7-methyl-3-methylene-oct-6-enoic acid (5). Their formation involved a sequence of oxidation reactions of the terminal double bonds by microsomal cytochrome P450 2B and epoxidation of the 1,2- and 3,10-epoxide intermediates, with subsequent hydrolysis to diols. This CYP-catalysed reaction was inhibited by several non-specific inhibitors of cytochrome P450 preventing from conversion of β-myrcene into 10-hydroxylinalool.

### Synergistic Effects of β-Myrcene With Other Active Natural Products

Studying the synergistic effect of monoterpenes, is important for determining the qualities present in different plant varieties. One study suggested that monoterpenes, including, β-myrcene found within a plant with other terpenes, may generate synergistic interactions (71). For example, β-myrcene can contribute to the overall flavour of beer due to its synergistic effect with other hop essential oils, such as, linalool. Other volatile compounds such as limonene, 3-carene and caryophyllene have been found to have an important role in the aroma and flavour of beverages and food products (72). There is also possibility of synergy between these compounds and β-myrcene, particularly if they share similar structure and notes (73, 74).

β-Myrcene contained within the cannabis plant may potentiate the innate anti-nociceptive properties of cannabinoids by lowering resistance across the blood brain barrier (BBB) and improving permeability, leading to an increase in transportation of cannabinoids into the brain (18). Additionally, the effect of β-myrcene as peripheral and central analgesics could be mediated to boost endocannabinoid derived central actions, when other terpenes are synergistically interacted with it (75). The terpenes were suggested to regulate the affinity of THC for CB1 receptor, which contributes to the improved analgesic effects of the cannabis plant (76, 77). Thus, one may be able to see a higher level of effects, rather than using an isolated component itself.

β-Myrcene found within the cannabis plant possesses anti-inflammatory, analgesic and sedative activities, which is additional to the effects of classical phytocannabinoids and may generate synergistic interactions (77, 78). β-Myrcene may act in synergy with tetrahydrocannabinol and other cannabinoids, such as CBD to enhance cannabis activity and eventually increase its psychoactive potential (77). However, a recent study suggested that cannabis-derived terpenoids functional effects were not detected, either alone or when combined with Δ9-tetrahydrocannabinol and cannabidiol (79). Thus, their ability to produce entourage activity by direct effects at cannabinoid receptors cannot be fully determined. The study concluded that none of the tested terpenes present in the cannabis plant (β-myrcene, pinene, caryophyllene and limonene) has a direct interaction with CB1 or CB2 receptors. Also, there were unaltered modifications to the activity of Δ9-tetrahydrocannabinol and cannabidiol. This study is in agreement with a previous study conducted by Santiago and his colleague, in which they rule out the direct activation of CB_1_ or CB_2_ or modulating the signalling of the phytocannabinoid agonist Δ9-tetrahydrocannabinol by β-myrcene in cannabis plant (80). However, both studies cannot rule out the existence of an entourage effect of β-myrcene as they only examined cannabinoids signalling through one pathway. There are possibility of entourage effects emerging through the impact of terpenoids on other pathways of endocannabinoid system or through non-cannabinoid receptor mechanisms that are important for the behavioural effect of *Cannabis* strains (81, 82).

## Purity of Commercially Available Myrcene

β-Myrcene is available commercially in an untreated technical grade (purity, 75%). High-purity β-myrcene (purity, >90%) is extracted using rectification (2). Impurities in β-myrcene are mainly other monoterpenes including: β-pinene, limonene, *dl*-limonene and *psi*-limonene. Dipeptine from a cyclization reaction and isomers and dimers of β-myrcene have also been observed (2).

Polymerization inhibitors are chemical compounds added to high purity β-myrcene to prevent their auto-polymerisation and to prolong its shelf-life (26). If β-myrcene is stored at 3°C, there is no loss by polymerisation for up to 12 months without an inhibitor. Most commercial food products containing myrcene, have polymerisation inhibitors such as α-tocopherol (2).

## Natural Occurrences of β-Myrcene

β-Myrcene is a component of the hydrocarbon fraction of many essential oils (83). It occurs naturally in over 200 plants and is present in the emissions of many trees in different parts of the world (26). Exposure to β-myrcene from natural food sources, is estimated to be 16,500 times more than from its synthetic use as a flavour substance (8).

The concentrations of β-myrcene in essential oils varies considerably between different plant species and varieties as well as plant parts (botanical drugs) (84). It can be found in significant quantities in the essential oils of hops and cannabis. Supplementary Tables 1–3 supply an overview of the relative concentrations of β-myrcene in essential oils and food products. The highest content of β-myrcene was found in Hops (Maximum: 10 g/kg dry weight) (3). The final concentration of β-myrcene in beer (0.4–80 μg/L) is much lower than in hops (52, 58). This is possibly due to dilution, variable extraction methods and it being destroyed by heating processes (58).

Quantification of β-myrcene from *C. sativa* extract has been studied in different varieties of *C. sativa* (85, 86). A more comprehensive description can be found in a recent study by Ibrahim et al. (45), which examined three varieties of *C. sativa* (45). One variety has high THC content (HP), the other one with high CBD content (HD), and the last was an intermediate variety containing both THC and CBD at a significant level. β-Myrcene content was higher in the intermediate variety than the other two varieties (0.87–1.32 mg/g). In the HD variety, β-myrcene content was 0.54–0.68 mg/mL compared to 0.19– 0.72 mg/g in HP varieties. Thus, the observations from this study may help in differentiation and the selection of specific variety of *C. sativa* based on their β-myrcene content.

Variations also exist between different geographical areas, season of harvesting, part of the plant and agronomical factors in different essential oils (22, 87). GC/MS analysis has shown that differences in β-myrcene concentrations exist between the different life cycles of a plant (vegetative and flowering) (84). Additionally, distillation periods and extraction methods can influence the yield of β-myrcene (88, 89).

## Important Pharmacological and Biological Effects of β-Myrcene

In the following we discuss some of the most salient pharmacological and biological effects (Supplementary Table 4).

### Central Nervous System Effects and Neurobehavioral Activity

Myrcene is well-known for its anxiolytic and sedative effects, which are both desired therapeutic actions, but may also pose some risks. Sedating agents can create drowsiness and impair motor coordination, and this can be assessed using the “rota-rod test” in animal studies measuring the length of time a rodent can balance on a rotating horizontal rod. A relatively high dosage of 200 mg/kg (1,468 μmol/kg) body wt myrcene, led to a 48% decrease in the time of permanence on the bar in the rota-rod test. The same dose of myrcene prolonged barbiturate sleep time 2.6 times. This was more intense in the presence of citral (20). Similarly, a single oral dose of β-myrcene, prolonged potentiated pentobarbital sleeping time, when administered 60 min before the barbiturate, possibly by inhibiting the barbiturates metabolism via cytochrome P450 (CYP) (78).

The main essential oil obtained from *Cannabis sativa* L. (Cannabaceae; hemp) (myrcene content: 22.9%), demonstrated measurable effects on the autonomic nervous system in healthy human subjects (*n* = 5). Inhalation of cannabis essential oil for 5 min improved nerve activity and was shown to relive stress and anxiety (Sweet almond oil was used as a control). The subjects generally felt more relaxed, energetic, calm, and an elevated mood, five min post inhalation. The study also used an electroencephalogram (EEG) to measure brain activity and results showed that there was an increase in theta (4–8 Hz) and alpha (8–13 Hz) brain wave activity in the posterior brain region, which is comparable to the EEG waves of individuals undergoing meditation (90).

Myrcene has also been shown to function as an anticonvulsant agent. Myrcene, obtained from *Lippia alba* (Mill.) N.E.Br. ex Britton and P.Wilson increased the latency of pentylenetetrazol-induced (PTZ) convulsions and increased the percentage of survival in female Swiss mice (91). Similarly, essential oils from *Cinnamosma madagascariensis* Danguy (8.9% myrcene) was evaluated *in vivo* for its anticonvulsant effects in Wistar rats that underwent induced seizures using pentylenetetrazole (PTZ). The study demonstrated the antiepileptic potential, by attenuating convulsions with moderate sedative effects. The possible mechanism of action was linked to glutamatergic and GABAergic neurotransmission (92). On the other hand, Da-Silva et al. (93) were unable to demonstrate the protective role of myrcene against PTZ-induced seizures. They were also unable to demonstrate benzodiazepine-like anxiolytic activity and the anti-depressive and antipsychotic effects of myrcene (93).

β-Myrcene may have significant clinical potential in adjuvant therapies, both as a pure compound and as a part of extract preparations. Popular anxiolytic essential oils are rich in other terpenoid alcohols, such as linalool, geraniol and citronellol, which might work synergistically with β-myrcene. Due to limited studies in human participants, small sample sizes, short duration of β-myrcene application, limited diverse administration of β-myrcene, the potential beneficial effect of β-myrcene on neurological disorders need further and more rigorous assessment.

### Antioxidant Activity

Antioxidant agents are accountable for the prevention of ageing and degenerative diseases such as atherosclerosis, cardiovascular diseases, cancer, diabetes and neurological illnesses (94). They also have an important role in inhibiting lipid oxidation within food products. In recent decades, there has been growing interest in the use of naturally occurring antioxidants in food preservation (22).

Selected monoterpenes have been studied for their potential antioxidant capacities, which can be attributed by the presence of conjugated double bonds that create chain breaking antioxidant activity. Chemical antioxidant assays like the DPPH assay (22, 95), are excluded here since they are of no pharmacological relevance. There is no evidence for therapeutic benefits on the basis of such chemical assays (96).

*In vivo* myrcene (97–100) demonstrate some relevant antioxidant effects. Ciftci et al. treated female Sprague-Dawley rats exposed to the environmental contaminant 2,3,7,8-tetracholorodibenzo-p-dioxin (TCDD) with myrcene [with a high dose of up to 200 mg/kg bw per day (1,468 μmol/kg) for 30 or 60 days]. These rats had a decrease in hepatic lipid peroxidation via activation of antioxidant and radical scavenger properties (97). Myrcene, again at a high dose of 200 mg/kg (1,468 μmol/kg) played a neuroprotective role in cerebral ischemia/reperfusion injury in C57Bl/J6 mice. In addition, myrcene increased glutathione along with other antioxidant enzymes such as glutathione peroxidase (GPx) and superoxide dismutase, thereby preventing oxidative damage and protecting brain tissue (99).The effects of orally administered β-myrcene (7.5 mg/kg bw; 55 μmol/kg bw) against ethanol-induced gastric ulcers in male Wistar rats is mediated through antioxidant effects via increased levels of GPx, glutathione reductase (GR) and total glutathione in gastric tissue (98). Importantly, future studies investigating antioxidant activity of β-myrcene, need to use appropriate dosage levels that have therapeutic effects in humans. This requires a comprehensive investigation into the recommended dosage of β-myrcene in humans. Additionally, most studies in the literature utilise chemical assays to detect antioxidant activity of β-myrcene. Future studies should use pharmacologically relevant *in vivo* or cell-based models to measure antioxidant activity.

### Anti-ageing Activity

As myrcene is an effective antioxidant compound, it may play a protective role against UVB-induced human skin photo-ageing. UVB exposure is associated with an overproduction of reactive oxygen species (ROS), which is a primary factor in oxidative skin damage (101). Abnormal production of ROS, activates numerous cell surface cytokines, growth factor receptors and mitogen activated protein kinases (MAPKs) (102). Exposure to UV radiation has also been shown to activate matrix metalloproteinases (MMPs), leading to the atrophy of collagen and elastin fibres (103). To date, only one study has investigated the role of β-myrcene and anti-ageing. Myrcene ameliorated skin ageing via decreased production of ROS, MMP-1, MMP-3, interleukin-6 (IL-6) and increased transforming growth factor type 1 (TGF-1) and type I procollagen secretions in UVB-irradiated human dermal fibroblasts. β-Myrcene treatment also downregulated phosphorylation of MAPK-related signalling molecules. Thus, myrcene may have a vital role against age-associated skin oxidative damage in skin care products (104).

### Anti-inflammatory Activity

*In vitro* β-myrcene is a powerful anti-inflammatory agent. Its ability to lessen inflammation occurs via prostaglandin E-2 (PGE-2) (105). In a study by Souza et al. (106) myrcene was shown to be effective in inhibiting the inflammatory response induced by lipopolysaccharide, including cell migration (leucocytes, neutrophils, mononuclear macrophages and eosinophils) and production of nitric oxide in mouse models of pleurisy. A significant inhibition of **γ**-interferon and interleukin (IL)-4 was also observed (106). Male Wistar rats with isoproterenol induced heart failure showed signs of cardiac function abnormalities. On the other hand, rats who were pre-treated with myrcene were protected from cardiac failure (*p* < 0.001) and inflammatory signals were abrogated. β-Myrcene was involved in suppressing fibrotic markers such as matrix metalloproteinases (MMP-2 and MMP-9) and regulating the expression of inducible nitric oxide synthase (iNOS), Transforming growth factor beta (TGF-β) and miRNA (profibrotic agents) with potential future benefits in treating cardiac failure (107).

In an *in vitro* cartilage degradation model of osteoarthritis, myrcene (25–50 μg/mL; 183.5–367 μmol/kg) showed anti-inflammatory and anticatabolic effects on human chondrocytes. Cartilage degradation and osteoarthritis progression was slowed down. Myrcene decreased interleukin IL-1β-induced nuclear factor-κB (NF-κB) and jun terminal kinase (JNK). It further decreased ERK1/2, p38 activation and the expression of inflammatory iNOS. Myrcene decreased catabolic responses (matrix metalloprotease MMP1 and MMP13), whilst increasing the expression of anticatabolic genes (tissue inhibitor of metalloproteases TIMP1 and TIMP3). Additionally, myrcene decreased the expression of non-cartilage specific collagen I induced by IL-1β, thus promoting the maintenance of the differentiated chondrocyte phenotype (23).

The anti-inflammatory activity of β-myrcene may not only be credited to its antioxidant potential, but also with its interaction with signal pathway cascades involving cytokines and transcription factors. Thus, plant oils rich in β-myrcene could serve as an option help to alleviate anti-inflammatory diseases and their symptoms.

### Antinociceptive Activity

β-Myrcene has shown central and peripheral analgesic effects (108). Intraperitoneal administration of β-myrcene (10 mg/kg; 73 μmol/kg) provided antinociception in mice who underwent tests of acute pain (19). This effect was antagonised centrally by previous administration of naloxone (opioid antagonist) and yohimbine (α_2_ adrenergic antagonist), implying the role of the opioid and noradrenergic systems. The results imply that the antinociceptive effect is mediated by the release of endogenous opioids through the a_2_-adrenoreceptors (19). In addition, the peripheral sites were antagonised by inhibitors of nitric oxide synthesis (109).

Lemongrass essential oil (15–20% β-myrcene) presented strong analgesic effects similar to peripheral-acting opioids, when assessed under different experimental models of pain in rats. Unlike morphine, no tolerance was observed after 5 days of repeated dosing in rats (105).

β-Myrcene may play a significant role in treating pain through interaction with transient receptor potential cation channel subfamily V member 1 (TRPV1) channels (110) involved in peripheral nociception (detection of noxious heat and pain) (111). On the other hand, a more recent study was unable to confirm the result of terpenoids being able to activate TRPV1 channels, suggesting that additional molecular targets must be explored (112).

## The Safety of Myrcene

In the following we discuss the safety of β-myrcene (Supplementary Table 5).

### Adverse Skin Reactions

In a European multicentre study, of 1,511 consecutive dermatitis patients, only one patient reacted adversely to 3% β-oxidised myrcene (containing 30% of β -myrcene) (113), this indicates that myrcene is hypoallergenic on the skin and is safe for topical use. Undiluted β-myrcene was moderately irritating to rabbit skin (114); but was neither irritating nor sensitising after being tested at 4% (*n* = 25) (115). β-Myrcene (5%) was sensitising to two of eleven patients sensitive to tea tree oil (116).

### Acute Toxicity

In mice and rats, the acute oral toxicity of β-myrcene was low, with an approximate lethal dose (ALD) of >5.06 g/kg bw (37,143 μmol/kg bw) and 11.39 g/kg bw (83,609 μmol/kg bw), respectively. Administration of β-myrcene via intraperitoneal injection had a lower ALD in mice and rats (2.25 g/kg bw and 5.06 g/kg bw, respectively; 16,516 μmol/kg bw and 37,143 μmol/kg bw, respectively), which is likely due to drug- induced chemical peritonitis (117). The acute oral LD_50_ in rats and the acute dermal LD_50_ in rabbits were reported to exceed 5 g/kg body weight (36,703 μmol/kg bw), following oral administration and dermal application (114).

### Subacute and Sub-chronic Toxicity

In a 14 week gavage study (Good Laboratory Practise compliant), male and female F344/N rats and B6C3F1 mice (10/group) were given doses of β-myrcene at 0.25, 0.5, 1, 2, or 4 g/kg bw (1,835, 3,670, 7,341, 14,681, 29,362 μmol/kg bw) for 5 days per week (human equivalent daily dose range: 17.5–280 g) (26). All 4 g/kg (29,362 μmol/kg) mice and rats died within 2 weeks, with other deaths observed in groups administered >0.5 g/kg (>3,670 μmol/kg). At the end of the 14 weeks, renal tubule necrosis significantly increased in rats of each dosage groups (not tested in mice). In rats with a dosage >1 g/kg (>7,341 μmol/kg), increased chronic inflammation, inflammation of the forestomach, mesenteric lymph node atrophy and olfactory epithelium degeneration was observed (26).

In a 90 day toxicity study utilising groups of male and female Sprague Dawley rats (10/sex and group), at the request of the EFSA, β-myrcene was administered in a diet containing 0, 700, 2,100, or 4,200 ppm of β-myrcene daily designed to provide targeted doses of 50, 150, or 300 mg/kg bw/day (367, 1,101, 2,202 μmol/kg bw/day) (118). No effects on mortalities, clinical signs of toxicity, haematology and clinical chemistry parameters and organ weights in the presence of β-myrcene within the diet was reported. Furthermore, the histopathological findings observed were not related to ingestion of β-myrcene and were either incidental or spontaneous. The oral NOEL for both sexes of rats, was the highest dose tests: 115 and 136 mg/kg bw/day (844 and 998 μmol/kg bw/day) for males and females (118). It should be noted that this calculated NOEL, is several orders of magnitude greater than human exposures from β-myrcene (119).

### Reproductive Toxicology

In a 3 month Gavage Study of β-myrcene, no effects on the weight of reproductive organs, sperm count or oestrous cyclicity was observed in doses of up to 2 g/kg (14,681 μmol/kg) in rats and up to 1 g/kg (7,341 μmol/kg) in mice (26). Administration of high doses of β-myrcene (1,200 mg/kg bw/day; 8,809 μmol/kg bw/day) on days 6–15 of pregnancy, was found to induce embryofoetal toxicity in pregnant Wistar rats. High doses decreased maternal body weight gain, increased the incidence of foetal skeletal malformations, lowered the number of visible implantation sites and the number of live foetuses. Additionally, foetal weights were lower, in comparison to the control group. The no-observable-adverse-effect level (NOAEL) of oral administration of β-myrcene for maternal and offspring toxicity was 500 mg/kg bw/day (3,670 μmol/kg bw/day) (67).

Two addition studies of β-myrcene on reproductive and development toxicity, have also indicated the adverse effects of β-myrcene on birth weight, peri and post-natal mortality, as well as foetal developmental abnormalities in Wistar rats (120, 121). The NOELS for fertility and general reproductive performance has been estimated as 250 mg/kg bw (1,835 μmol/kg bw) (120) and 300 mg/kg bw (2,202 μmol/kg bw) (121). No data on the reproductive or developmental toxicity of β-myrcene in humans is currently available.

### Mutagenicity and Genotoxicity

Myrcene inhibited cyclophosphamide induced sister-chromatid exchanges in Chinese hamster V79 cells and cultured hepatic tumour cells (122). Myrcene had no genotoxic potential in mammalian cells *in vitro* and did not induce chromosome aberrations or sister-chromatid exchange. β-Myrcene reduced CP-induced sister-chromatid exchanges in human lymphocytes in a dose dependent manner. Also, it did not influence the genotoxicity of methane sulfonate and benzo[a]pyrene (123). Additionally, β-myrcene (doses ranging from 100 to 1,000 μg/mL; 734 to 7,341 μmol/kg) reduced the cytotoxic and mutagenic effects of CP in V79 Chinese hamster cells, when tested with rat liver S9. The authors suggested that myrcene had the ability to inhibit cytochrome P-450 isoenzymes which activates compounds with mutagenic and carcinogenic properties (123).

No evidence of chromosomal aberrations in bone marrow cells of rats administered β-myrcene (0.1, 0.5, or 1.0 g/kg bw; 734, 3,670, 7,341 μmol/kg bw) was observed. Although there was no evidence of myrcene-induced clastogenicity, there was a dose-dependent increase in the mitotic index of bone marrow cells at 24 h (124). Additionally, there was no increase in the frequency of micronucleated normochromatic erythrocytes, a biomarker of both acute and cumulative chromosomal damage, in B6C3F1 mice treated with β-myrcene (0.25 to 2 g/kg; 1,835 to 14,681 μmol/kg) by gavage for 3 months (26).

β-Myrcene expressed antimutagenic activities against aflatoxin B_1_ (AFB_1_) in *Salmonella typhimurium* (TA100). Doses of 1.5 and 3.0% of β-Myrcene, showed inhibitory actions of 65 and 73%, respectively when tested with 1.0 μg/plate AFB_1_ in the presence of exogenous metabolic activation (rat liver S9) using TA100 and the pre-incubation method (125). The NTP (2010) and (126), concluded that β-myrcene was not mutagenetic based on the negative Ames test using Salmonella strains (TA97, TA98, TA100, and TA1535) with and without metabolic activation. It was also negative in the *Escherichia coli* test system (strain WP2 uvrApKM101) with and without metabolic activation (S9 fraction from Aroclor 1254-induced rat or hamster liver), and in an *in vivo* micronucleus assay in B6C3F1 mice (26).

β-Myrcene inhibited the activity of pentoxyresorufin-O-depenthylase (PROD), a selective marker for mono-oxygenase CYP2B1, necessary for the activation of genotoxins in rats (127). β-Myrcene also demonstrated protective effects against t-butyl hydroperoxide induced genotoxicity in human B lymphoid NC-NC cells, which was predominantly mediated by their radical scavenging activity (128).

### Carcinogenicity/Anti-carcinogenic Activity

Multiple studies have demonstrated that β-myrcene exposure had anticarcinogenic potential in *in vitro* models. Administration of β-myrcene suppressed the *in vitro* formation of N-Nitrosodimethylamine (NDMA), a potent carcinogen, by 88% (129). In MCF-7 cells, β-myrcene (IC_50_, 291 μM) inhibited the breast cancer cells growth *in vitro*, but was slightly toxic to normal Chang liver cells (IC_50_, 9.5 mM; 9,500 μmol/kg) (130). Additional studies have also implicated the cytotoxic effect of β-myrcene against a broad range of cancer cells, such as MCF-7 breast carcinoma, HT-29 colon adenocarcinoma (131), P388 leukaemia cells (132) and other tumour cell lines (25, 133, 134).

On the other hand, in a model of 7,12-dimethylbenz[a]anthracene (DMBA)-induced mammary carcinogenesis, β-myrcene did not exhibit significant chemopreventive activities. β-myrcene did not reduce the total number of mammary tumours or the median tumour latency period in a group of female Sprague-Dawley rats (age, 6 weeks) fed diets containing β-myrcene (purity, 94.3%) (135).

In the 2010 NTP study, a gavage study in groups of male and female F344/N rats and B6C3F1 mice was conducted over 2 years. Rats and mice were given doses of β-myrcene (0, 250, 500 or 100 mg/kg/day; 0, 1,835, 3,670, 734 μmol/kg/day) in corn oil for 5 days per week (26). The β-myrcene provided had a purity of 90% and contained other impurities, such as ψ-limonene, (±)-limonene and isomers and dimers of β-myrcene. The presence of other components could render the carcinogenic results attributed to myrcene in the NTP study as potentially invalid. The doses given to the rodents had a strength five orders of magnitude greater than the exposure to food flavouring additives containing β-myrcene, which is normally found in a human population (27).

The results of the NTP study showed that there was an increased incidence of renal tubule adenoma or carcinoma in treated male rats (not for female rats, or mice). Furthermore, there was clear evidence of a dose-dependent increase in hepatocellular adenoma, hepatocellular carcinoma, and hepatoblastoma in male B6C3F1 mice (26). Spontaneous tumours were also observed in the vehicle control group, this is of no surprise as the male B6C3F1 mouse is known for having a high background incidence of hepatocellular tumours and may not be of relevance to humans (27).

## Regulatory Positions

The U.S. FDA conducted a safety review of β-myrcene, following the submission of a Food Additive Petition (FAP 5A3810) by a coalition of NGOs (136). The petition requested the removal of the use of synthetic β-myrcene, as previously approved in the food additive regulations (21 CFR 172.515) (8, 137). The petition was based on the results of carcinogenicity studies undertaken in mice and rats treated with β-myrcene, from the National Toxicology Program (NTP) (26).

The FDA stated that β-myrcene did not demonstrate genotoxic potential and was unlikely to induce tumours in humans at its current exposure level as a food flavouring chemical (8). Although the FDA stated that β-myrcene did not pose a risk to the public health under conditions of intended use, it was removed from the food additive regulations under the Delaney Clause of the Federal Food, Drug, and Cosmetic Act in October 2019. This clause requires the Food and Drug Administration (FDA) to ban food additives which are found to cause or induce cancer in humans or animals as indicated by testing (137).

The EFSA stated that there are no safety concern relating to β-myrcene, including no evidence of a potential genotoxic or mutagenic activity (138). Additionally, the Expert Panel of the Flavour and Extract Manufacturers Association (FEMA) most recently evaluated the safety of 54 citrus derived natural flavour complexes. The FEMA panel confirmed that β-myrcene is “generally recognised as safe (GRAS)” (139). According to the Joint FAO/WHO Expert Committee on Food Additives (JEFCA), β-myrcene is safe to use as a flavour ingredient and is not of concern at its current estimated intake (140). More recently, the RIFM stated that is safe to use for fragrances, and that there is no evidence for genotoxic, skin sensitising, phototoxic/photoallergenic effects (141).

## Conclusion

β-Myrcene is an abundant compound which occurs naturally as a major constituent in many plant species. Despite it is being common practise to attribute the biological properties of an essential oil to β-myrcene, as with all herbal preparations, the role of a range of metabolites and the specific composition of an essential oil is a crucial parameter to be taken into consideration in such an assessment (142).

The many significant biological properties of β-myrcene coupled with its non-allergic, non-toxic and antimutagenic activities offers the possibility of incorporating this natural product into medical or cosmetic products. Botanicals, such as raw hops are rich in β-myrcene, and play an important role in enriching the aroma of beer (26). There is a growing trend for alcohol free functional drinks in the beverage sector, since health consciousness is rising (143). The use of non-alcoholic functional beverages could offer the interesting health-related properties of β-myrcene e.g., relaxing, stress reducing and sleep enhancing, without the negative effects of ethanol on the liver and other organs. Companies such as Three Spirit Drinks (Beyond Alcohol Ltd.), are currently promoting the use of β-myrcene in their functional non-alcoholic beverage formulations as well as developing novel methods to create further β-myrcene rich products. The suggested anxiolytic effects of myrcene may also lead to the development of more functional non-alcoholic hop based products that are able to provide the perception of relaxation without the harmful effects of alcohol. Additionally, subsequent to the international relaxation of marijuana prohibition legislation, breeding work has already resulted on Cannabis chemotypes producing 97% of monoterpenoid content as β-myrcene (77). Such a preparation may lead to novel approaches in treatment of numerous clinical conditions.

The significant biological role of β-myrcene in plant essential oils may be limited due to the existing chemical variability. Fluctuations of the chemical composition occur due to harvesting time, climate, age of plant, plant parts, and extraction methods used. There is also possibility that β-myrcene found within plant essential oils act synergistically with other components within the essential oil to enhance health benefits. Therefore, potential synergism and antagonism should be further studied.

At present the FDA no longer permits the use of pure β-myrcene as a flavouring agent due to a legal challenge based on the Delaney Clause. Currently, no data is available that correlates the therapeutic use of pure β-myrcene with health benefits in human participants. Most of the studies on health benefits of β-myrcene in this review were in animal models or cell culture. Few studies conducted in humans (*n* = 2) were found and these included humans inhaling plant essential oil extracts containing <25% of β-myrcene. Thus, more robust, randomised, controlled clinical trials/intervention studies are needed using pure β-myrcene preparations to evaluate and replicate its beneficial effects in humans.

The dosages applied in the NTP study were five-six orders of magnitude greater than human exposure and there are also doubts of the purity of the β-myrcene used, thus casting serious doubts on the relevance to humans. The NTP concluded that the renal tumours in the low dose group of male F344 rats, were possibly due to α2u-globulin nephropathy. This is not applicable to humans, as the protein α2u-globulin responsible for this effect in rodents is not present in humans. In contrast, the susceptibility of different strains of rats to renal carcinogenicity varies widely (27), with Bastaki et al., finding a lack of renal toxicity to β-myrcene when using Sprague-Dawley rats. In addition, the B6C3F1 male mice included in the NTP study are recognised for having a high and variable background incidence of hepatocellular tumours; on this basis the EFSA has rejected its relevance for human health (144). Furthermore, based on this review, numerous studies have indicated the anti-mutagenic and anti-metastatic effects of β-myrcene on several different cancer cell lines (25, 145), highlighting the positive effects of β-myrcene on cancer prevention.

Overall, the evidence reviewed here points to β-myrcene being safe if consumed at a level, as it is common for food use (estimated daily intakes for β-myrcene is 1.23 μg/kg bw/day for a 60 kg person). More in-depth studies of β-myrcene toxicity in human target-organs and the establishment of protective exposure limits are needed to enhance the safe and effective use of β-myrcene.

Many questions remain yet to be answered, and not just to clarify the mechanisms of activity β-myrcene exerts in the human body, but also with regards to adopting consistent methodological criteria for future clinical research. Importantly, it needs to be evaluated to what extent β-myrcene achieves and maintains concentrations required for affecting neuronal activity in the brain. Additionally, comparisons of the intensity and durability of β-myrcene with conventional medicine needs to be investigated. Furthermore, sufficient knowledge of the efficient extraction and analysis methods of β-myrcene would help in maximising β-myrcene extraction whilst retaining its organoleptic qualities. Overall, a wide range of interesting biological activities and biochemical modifications in healthy subjects are likely to emerge from future research on β-myrcene.

## Author Contributions

SS, GS, DL, and MH were responsible for the study conception. SS, FQ, and GS drafted the manuscript. SS, FQ, GS, DL, and MH provided data and sense checked data analysis and critically reviewed the manuscript. All authors contributed to and approved the final version of the manuscript.

## Conflict of Interest

GS and DL were employed by the company Beyond Alcohol Ltd (trading as Three Spirit Drinks). GS and DL are employed by Beyond Alcohol Ltd., who use plants containing β-myrcene in their products. This study has arisen out of an assessment of the compound's safety initially planned as a collaborative project between Beyond Alcohol Ltd., and UCL, which, however, was not possible due to the pandemic of 2020. SS, FQ, and MH have been advising Beyond Alcohol Ltd., on the pharmacology and safety of β-myrcene.
