# Supplementary material for: Myrcene—What Are the Potential Health Benefits of This Flavouring and Aroma Agent?
Source: Front Nutr. 2021 Jul 19;8:699666. doi: 10.3389/fnut.2021.699666 (PMC8326332; doi:10.3389/fnut.2021.699666)
Supplement: Supplementary file 1 [file Data_Sheet_1.pdf]

Supplementary Table 1: Relative concentrations of β-myrcene in essential oils with a reported concentration of >10.0% in at least one study

Product	Botanical name	Family	Essential oil Source	β-Myrcene content	Country/ origin	Reference
Angelica species	*Angelica* archangelica L.	Apiaceae Lindl.	Roots	5.4– 13.3%	Norway	(Ojala et al., 1986)
			Roots	6.0-46.8%	Finland/Kemi Lapland/ Inari Lapland/ Somerniemi	(Ojala et al., 1986)
			Roots	7.1%	Serbia	(Aćimović et al., 2017)
			Root 1-2 mm<br>Root 3-4 mm<br>Root >5 mm	2.13%<br>1.5%<br>5.87%	Italy	(Pasqua et al., 2001)

			Seeds	3.4-7.3%	Finland/ West Lapland/East Lapland/North Lapland	(Holm et al., 1997)
			Fruits	2.0-2.5%	Lithuania: Prienai District/ Svencionys District	(Nivinskiene et al., 2007)
			Shoots	Fresh shoots: 17.0%<br><br>Convectively dried shoots: 21.3%<br><br>Freeze dried shoots: 26.7%	Department of Vegetable and Medicinal Plants, Warsaw University of Life Sciences - SGGW, Warsaw	(Roslon et al., 2016)
	Angelica archangelica Aa subsp. *archangelica* var. *sativa*		Roots	3.15–4.62%	Finland	(Forsén, 1979)
			Fruits	2.4–3.2%	France/Le Havre, Thiais/cultivation	(Bernard, 2001)
	Angelica acutiloba (Siebold & Zucc.) Kitag		Root, stem and leaves	6.7–8.6%	Nantou, Taiwan	(Chen et al., 2014)

	Angelica gigas Nakai		petiole	6.38%	Rutgers University, New Brunswick, NJ, USA	(Park et al., 2003)
	Angelica dahurica (Hoffm.) Benth. & Hook.f. ex Franch. & Sav.		Root	5.5%	Beijing, China	(Tabanca et al., 2014)
African bluegrass	*Cymbopogon validus* (Stapf) Stapf ex Burtt Davy	Poaceae Barnhart	Aerial parts	Plants cultivated in the wild: 23.1 - 35.6%<br><br>Cultivated mature plants: 11.6-20.2%	Nyanga in the Eastern Highlands ofZimbabwe and Harare, Zimbabwe	(Chagonda et al., 2000b)
Baraúna	*Schinopsis brasiliensis* Engl.	Anacardiaceae R.Br.	Leaves	45.12%	Brazil	(Donati et al., 2015)
Bay (West Indian)	*Pimenta racemosa* (Mill.) J.W.Moore	Myrtaceae Juss.	Leaves	26.6%	Benin	(Ayedoun et al., 1996)
			Leaves	0.10-16.17%	Dominican Republic	(Tucker et al., 1991)
			Leaves	25.1%	Benin	(Philippe et al., 2012)
			Leaves	25.1% - 29.4%	Benin	(Alitonou et al., 2012)

			Leaves	11.7%	Venezuela	(Contreras et al., 2014)
	P. racemosa var. *grisea*		Leaves	0.71%	Dominican Republic	(Tucker et al., 1991)
	P. racemosa var. *hispanidensis*		Leaves	0.26%-4.72	Dominican Republic	(Tucker et al., 1991)
	P. racernosa var. *ozua*		Leaves	0.45-0.86%	Dominican Republic	(Tucker et al., 1991)
Blackcurrant bud	*Ribes nigrum* L.	Grossulariaceae DC.	Blackcurrant berries	1.9-16.0%	Dundee, Scotland	(Ruiz del Castillo and Dobson, 2002)
Bushy Matgrass	*Lippia alba* (Mill.) N.E.Br. ex Britton & P.Wilson	Verbenaceae J.St.-Hil.	Aerial parts	15.0%	Oriximiná, Pará State, Brazi	(Oliveira et al., 2006)
Byunggyul	*Citrus platymamma*	Rutaceae Juss.	Peel	22.17%	Korea	(Baik et al., 2008)
Cape May	*Coleonema album* (Thunb.) Bartl. & H.L.Wendl.	Rutaceae Juss.	Herbal parts	20.5%	South Africa	(Baser et al., 2006)
			-	14%	Western Cape, South Africa	(Fajinmi et al., 2019)

Celery Leaf	*Apium graveolens* L. var. *dulce*	Apiaceae Lindl.	Leaves and Stems	8.0 (raw stalk) –73.0 (boiled leaves)	Nagano Prefecture, Japan	(Kurobayashi et al., 2006)
Clausena anisata	*Clausena anisata* (Willd.) Hook.f. ex.	Rutaceae Juss.	Leaves	14.3%	Nghệ An Province, Vietnam	(Trung et al., 2014)
Dangyuja	*Citrus grandis* (L.) Osbeck	Rutaceae Juss.	Peel	22.65%	Korea	(Baik et al., 2008)
Dongjunggyul	*Citrus erythrosa*	Rutaceae Juss.	Peel	25.27%	Korea	(Baik et al., 2008)
Distichoselinum tenuifolium	*Distichoselinum tenuifolium* (Lag.) Garcia Martin & Silvestre	Apiaceae Lindl.	flowering umbels and ripe umbels- with mature seeds	47.7-84.6%	Algarve province (South Portugal)	(Tavares et al., 2010)
East African satinwood	*Zanthoxylum gilletii* (De Wild.) P.G.Waterman	Rutaceae Juss.	Young leaves	42.87%	Cameroon	(Jirovetz et al., 1999)
Frankincense	*Boswellia sacra* Flück *(α-pinene chemotype)*	Burseraceae Kunth	Gum resin	0–20.7%	Somalia	(Svoboda et al., 2001)
Hemp	*Cannabis sativa* L.	Cannabaceae Martinov	Flowering tops	31.1%	Switzerland	(MEINER and

						MEDIAVILLA, 1998)
Holy flax	*Santolina rosmarinifolia* L.	Asteraceae Bercht. & J.Presl	Aerial parts	0.3–15.5%	Madrid, Spain	(Palá-Paúl et al., 2001)
Hop*	*Humulus lupulus* L. var. *Cascade*	Cannabaceae Martinov	Inflorescence	25.4%	Rio Negro Province, Argentina	(Malizia et al., 1999)
Lavender cotton	*Santolina chamaecyparissus* L.	Asteraceae Bercht. & J.Presl	Fresh lobed, silver-grey leaves	15.0%	Marseilles, France	(Vernin, 1991)
			Aerial parts	6.44	North Eastern Algeria	(Zaiter et al., 2015)
Lemongrass	*Cymbopogon citratus* (DC.) Stapf	Poaceae Barnhart	Plant biomass	5.6–18.6%	Harare, Zimbabwe	(Chagonda et al., 2000a)
			Leaves	11.0	Ouagadougou, Burkina Faso	(Bassolé et al., 2011)
			Leaves	Not detected	Rio de Janeiro, Brazil	(Pinto et al., 2015)
			Leaves	6.52%	Holguín, Cuba	(Pinto et al., 2015)
Mango Ginger	*Curcuma mangga* Valeton & Zijp	Zingiberaceae Martinov	Air-dried rhizomes	46.50%	Malaysia	(Wahab et al., 2011)
Mastic	*Pistacia lentiscus* L. var. *chia*	Anacardiaceae R.Br.	Gum resin	4.72 -27.58	Chios Island, Greece	(Paraschos et al., 2016)

Mountain tea (chaye-kuhi)	*Stachys lavandulifolia* Vahl	Lamiaceae Martinov	Aerial parts	0.0–26.2%	Iran	(Aghaei et al., 2013)
Myrtle (honey)	*Melaleuca teretifolia* Endl. (Citral Chemovar.B)	Myrtaceae Juss.	Leaves and twigs	6.3-13.3%	Western Australia	(Southwell et al., 2003)
Needle juniper	*Juniperus rigida* Siebold & Zucc.	Cupressaceae Gray	Berries	27.0%	Yulin city, Shaanxi province,China	(Liu et al., 2016)
Juniper berry	*Juniperus communis* L.	Cupressaceae Gray	Fresh ripe (black) and unripe (green) berries, small and big shrub	4.8–19.6%	Vilnius, Lithuania	(Butkienë et al., 2004)
Parsley leaf	*Petroselinum crispum* (Mill.) Fuss	Petroselinum crispum (Mill.) Fuss	Leaves	2.4–13.8%	Greece	(Petropoulos et al., 2004)
Pepper (pink)	*Schinus molle* L.	Anacardiaceae R.Br.	Fruits	26.4 – 42.0%	Yaso, Peru	(Huaman et al., 2004)
				8.4–12.8%	North-eastern Tunisia	(ZAHED et al., 2011)
Pepper (Sichuanese)	*Zanthoxylum bungeanum* Maxim.	Rutaceae Juss.	Air-dried ripe pericarp	3.59- 17.50%	Yangling, Shaanxi Province, China	(Liu et al., 2017)

Pine (white)	*Pinus strobus* L.	Pinaceae Spreng. ex F.Rudolphi	Needles (leaves)	5.8-16.2%	Ottawa, Canada	(Rudloff, 1985)
Pomelo	*Citrus grandis* (L.) Osbeck	Rutaceae Juss.	Peel	22.81–30.93%	China	(Shao et al., 2014)
Pteronia	*Pteronia incana* (Burm.) DC.	Asteraceae (Compositae)	Aerial parts	10.3%	Eastern Cape, South Africa	(Bruns and Meiertober ens, 1987)
Pyungyul	*Citrus tangerina Hort.* ex Tanaka	Rutaceae Juss.	Peel	32.10%	Korea	(Baik et al., 2008)
Sweet wormwood	*Artemisia annua* L.	Asteraceae Bercht. & J.Presl	Flowers	0.20–37.71%	Chongqing, China	(Yu et al., 2011)
Tansy (blue)	*Tanacetum annuum* L.	Asteraceae Bercht. & J.Presl	Aerial parts	1.1–13.8%	North Morocco	(Greche et al., 1999)
Temu pauh	*Curcuma mangga* Valeton & Zijp	Zingiberace ae Martinov	Dried rhizomes	46.5%	Malaysia	(Wahab et al., 2011)
Thymus kotschyanus	*Thymus kotschyanus* Boiss. & Hohen.	Lamiaceae Martinov	Leaves	0.26-12.65%	Damavand area of Iran	(Rasooli and Mirmostafa, 2003)
Thymus serpylloides	*Thymus serpylloides* Bory ssp. gadorensis	Lamiaceae Martinov	Aerial parts	0.13–30.39	South East of Spain	(Sáez, 2001)
Thymus serpyllum	*Thymus serpyllum* L.	Lamiaceae Martinov	Whole dried	0–20.2%	Estonia, Russia,	(Paaver et al., 2008)

					Latvia and Armenia	
Wild carrot	*Daucus carota* L.	Apiaceae Lindl.	Seeds, flowers and fruits	0.5–10.5%	Italy	(Flamini et al., 2014)
Yuxingcao	*Houttuynia* Thunb*.*	Saururaceae F.Voigt	Fresh plants	2.58—18.47%	Huaihua (central China)	(Lu et al., 2006)

* - the concentration of hop products are highly variable, see **supplementary table 1**

Supplementary table 2: Percentage compositions of myrcene essential oils in selected hops

Variety	Myrcene (%)	Description of Hop[a]	Reference
Amarillo	23.3 - 39.5	Orange, Peach, Pink Grapefruit	(Duarte et al., 2020)
Brewers Gold	63.0	Blackcurrant, Lemon, Spicy	(Guadagni et al., 1966)
Cascade	48.9	Floral, fruity, and particularly citrusy (grapefruit), with little earthy or spicy aroma	(Nance and Setzer, 2011)
	21.8		(Duarte et al., 2020)
Citra	44.3	Grapefruit, Lime, Mango	(Duarte et al., 2020)
Cluster	40.3 – 49.4	Floral, Spicy, Blackberry	(Eri et al., 2000)
Columbus	29.8-31.7	Liquorice, Resinous, Black Pepper	(Duarte et al., 2020)
El dorado	31.6	Melon, Peaches, Pineapple	(Duarte et al., 2020)
Fuggle	3.8 -10.8	Earthy, Grassy, Minty	(Duarte et al., 2020)
Galena	32.1 – 32.6	Blackcurrant, Grapefruit, Spicy	(Eri et al., 2000)
Hallertauer	21.4-24.8	Mild, yet spicy, with floral and citrus tones	(Nance and Setzer, 2011)
Herkules	21.6	Pine, Spicy, Black Pepper	(Duarte et al., 2020)
Hersbrucker	4.9	Earthy, Floral, Herbal	(Duarte et al., 2020)
Magnum	29	Floral, Herbal, Pine	(Duarte et al., 2020)
Mandarin Bavaria	5.3 – 10.4	Lemon, Spicy, Mandarin	(Raut et al.)
Marynka	74.1–89.3 (wt%)	Floral, Herbal, Lemon	(Tyśkiewicz et al., 2018)
Mittelfruh	11.2	Floral, Grassy, Herbal	(Duarte et al., 2020)

Mosaic	45.3	Mango, Passionfruit, Blueberry	(Duarte et al., 2020)
Northern Brewer	52.4	Medium intensity, pine and mint characteristics	(Nance and Setzer, 2011)
	27.90		(Duarte et al., 2020)
Nugget	28.3 – 31.8	Herbal, Spicy, Pear	(Eri et al., 2000)
	33.8		(Duarte et al., 2020)
Perle	6.9	Cedar, Orange, Spicy	(Duarte et al., 2020)
Saaz	8.0-25.7	Mild spice, cinnamon-like and earth tones	(Nance and Setzer, 2011)
	53.0		(Gonçalves et al., 2012)
	5.2 -13.1		(Duarte et al., 2020)
Simcoe	34.2	Grapefruit, Passionfruit, Pine	(Duarte et al., 2020)
Sterling	35.8	Spicy and herbal with a little floral aroma	(Nance and Setzer, 2011)
Target	5.5	Cedar, Orange, Pine	(Duarte et al., 2020)
Tettnang	13.1	Earthy, Floral, Herbal	(Duarte et al., 2020)
Tradition	5.1	Floral, Grassy, Herbal	(Duarte et al., 2020)
Vanguard	16.8	Herbal and floral tones	(Nance and Setzer, 2011)
Wild hop ecotype	26.9		(Paventi et al., 2020)
Willamette	39.8	Earthy, slightly spicy, fruity, and flora	(Nance and Setzer, 2011)
	27.6 – 30.1		(Eri et al., 2000)

[a] Description of Hops is taken from: https://www.charlesfaram.co.uk/

Supplementary table 3: Relative concentrations of β-myrcene in selected food products

Product	Myrcene concentration	Country or region	Reference
Hops oil*	479 mg/L	Germany	(Van Opstaele et al., 2012)
Hops*	5489 µg/g dw (Average concentration)	USA	(Aberl and Coelhan, 2012)
Alcoholic beverages	Mean= 1.12 mg/L<br>Maximum= 5.00 mg/L	USA	(Burdock, 2019)
Beer	45.6–79.7 µg/L	USA and Germany	(Schmidt and Biendl, 2016)
Beer	0.49-0.56 µg/L	Czech Republic	(Mikyška and Olšovská)
Baked goods	Mean= 10.05 mg/kg<br>Maximum= 14.92 mg/kg	USA	(Burdock, 2019)
Carrots (Daucuscarota L.) of cv. Bolero and cv. Carlo	80.0–219.0 ng/g	Denmark	(Kjeldsen et al., 2003)
Chewing gum	Mean= 116.2 mg/kg<br>Maximum= 126.00 mg/kg	USA	(Burdock, 2019)
Condiments, relishes	Mean= 5.00 mg/kg<br>Maximum= 10.00 mg/kg	USA	(Burdock, 2019)
Fennel fruits	1150 µg/g	Hungary	(Zeller and Rychlik, 2006)
Fennel tea (prepared)	140 µg/L	Hungary	(Zeller and Rychlik, 2006)
Frozen dairy	Mean= 12.32 mg/kg<br>Maximum= 15.68 mg/kg	USA	(Burdock, 2019)
Gelatine, puddings	Mean= 19.96 mg/kg<br>Maximum= 22.91 mg/kg	USA	(Burdock, 2019)
Italian lemon liquors (Limoncello)	3.6 –31.0 mg/L	Italy	(Andrea et al., 2003)
Mango (cultivar 'Haden')	65.9 µg/kg	USA	(Munafo et al., 2016)
Meat products	Mean= 5.00 mg/kg<br>Maximum= 10.00 mg/kg	USA	(Burdock, 2019)
Non-alcoholic beverages	Mean= 7.72 mg/L<br>Maximum= 11.15 mg/L	USA	(Burdock, 2019)

Pomegranates	0.01 g/kg	Spain	(Calín-Sánchez et al., 2011)
Soft candy	Mean= 6.22 mg/kg<br>Maximum= 8.07 mg/kg	USA	(Burdock, 2019)

* **-** the concentration of hops products is highly variable, see **supplementary table 2**

Supplementary table 4: β-myrcene mechanisms of action

Pharmacological activity assessed	(Essential oil (EO) or Constituent) as reported in the original source (>5% included)	Study Type	Experimental procedures	Key findings	References
Anxiolytic	Cannabis EO (**22.9% β-myrcene**); *Cannabis sativa* L.	Placebo-controlled studies (Human)	Five healthy volunteers (3 males and 2 females) aged 30 to 57 years were recruited for the study.<br>• Participants were asked to inhale 1 mL of sweet almond oil (control) for 5 minutes.<br>• After a 5-minute break, participants inhaled 1 mL of Cannabis EO (THC <0.2% w/v) for 5 mins.	• Subjects described themselves as more energetic, relaxed, and calm.<br>• The brain wave activity and autonomic nervous system are affected by Cannabis sativa essential oil inhalation suggesting a neuromodular activity in cases of stress, depression, and anxiety.	(Gulluni et al., 2018)
Anxiolytic	Petitgrain EO (**1.3–12.12% β-myrcene**); *Citrus aurantium* ssp. *amara*	Placebo-controlled trial (Human)	42 administrative university workers (Mean age = 42.21 years, 10 male).<br><br>The participants were randomly assigned into a petitgrain EO group (AG) and a control group	• The AG performed the Web site typing task 2.28 min faster than the CG (p = 0.05), suggest improvements in arousal levels.<br>• An increase in parasympathetic activity for the AG between the Pre-test and during the intervention was observed. There was also a decline in	(Huang and Capdevila, 2017)

			containing neutral almond oil (CG).<br><br>At the same time, participants completed a computer task on a specific Web site typing on their keyboard until they had finished it.	sympathetic activity for the AG group, suggests the role of the oil on stress reduction.	
Anxiolytic	• Lavender oil (**5.3% β-myrcene**); *Lavandula officinalis* Chaix<br>• Synthetic β-myrcene (**purity unknown)**	In vivo (mice)	Male ICR mice were injected with lavender oil and synthetic β-myrcene which were diluted in olive oil. The mice were injected intraperitoneally (1ml/100 g bw). The mice were then investigated with two conflict tests: Geller and Vogel.	• Lavender oil produced significant anticonflict effects at 800 and 1600 mg/kg in the Geller conflict test.<br>• Lavender oil produced significant anticonflict effects at 800 mg/kg in the Vogel conflict test, suggesting that the oil has an anti-anxiety effect.<br>• β-Myrcene did not produce any significant anticonflict effects in the Geller test.	(Umezu et al., 2006)
Anxiolytic	• Rose oil (**Percentage of β-myrcene is unknown**); *Rosa centifolia*<br>• Synthetic β-myrcene (**purity unknown)**	In vivo (mice)	Male ICR mice were injected with rose oil and synthetic β-myrcene which were diluted in olive oil. The mice were injected intraperitoneally (1ml/100 g bw).<br><br>The mice injected with rose oil were investigated with two conflict tests: Geller (N=10) and Vogel (N=15-18).	• Rose oil produced significant anticonflict effects in both the Geller and Vogel conflict tests. However, the cost of developing rose oil is too expensive.<br>• β-Myrcene did not produce any significant anticonflict effects in the Geller test and Vogel conflicts tests.	(Umezu et al., 2002)

			The mice injected with β-myrcene were then investigated with two conflict tests: Geller (N=17) and Vogel (N=18).		
Relaxant	• *Plectranthus barbatus* Andrews EO (**12.4% β-myrcene**)<br>• Synthetic β-myrcene (**purity is unkown**)	In vitro (animal)	*Plectranthus barbatus* EO at concentrations ranging from 1 to 300 microg/mL and some major constituents, e.g.., myrcene (0.1 - 30 µg/mL) were studied on the contractility of the guinea-pig ileum.	• The essential oil and α-pinene, had powerful direct relaxation effects on the guinea pig ileum. Spasms which were induced by specific and unspecific stimuli, were reversibly blocked and tissues with artificially increased tonus were relaxed.<br>• There was only a slight direct relaxant effect on intestinal tonus by myrcene (2.7 ± 0.8%).	(Câmara et al., 2003)
Relaxant	Synthetic β-myrcene (**purity is 90%)**	In vivo (mice)	Male Swiss mice were injected intraperitoneally with β-myrcene (50, 100 or 200 mg/kg body wt) for the open-field test, rota rod test and pentobarbital-induced sleeping time test. In the levated plus maze test myrcene was injected in mice at doses of 5, 10, 25 or 50 mg/kg body wt., i.p..	• In the open field test, myrcene (100 and 200 mg/kg body wt) decreased the number of crossing and numbers for rearing and grouping in the open field test.<br>• Muscle relaxation was detected at the highest doses of myrcene (100 and 200 mg/kg body wt.) in the rota rod test.<br>• Myrcene (100 and 200 mg/kg body wt.) increased barbiturate sleeping time as compared to control.<br>• In the elevated-plus maze test myrcene showed a dose-dependent effect, and this	(Gurgel do Vale et al., 2002)

				• effect was significant at the doses of 10 and 25 mg/kg.<br>• Overall, β-myrcene presented sedative and motor-relaxant effects.	
Relaxant	*Lippia alba* (Mill.) N.E.Br. ex Britton & P.Wilson, β-myrcene chemotype (Type I) (**percentage of β-myrcene is unknown**)	In vivo (mice)	Female Swiss mice were administered with β-myrcene intraperitoneally or orally 30 or 60 mins before the experiments at doses of either: 100 mg/kg, 200 mg/kg, 400 mg/g	• β-Myrcene was linked to an Increased seizure latency and percentage of survival, as compared to controls.	(Viana et al., 2000)
Neurobehavioral	Lemongrass tea (**percentage of β-myrcene is unknown**); *Cymbopogon citratus* (DC.) Stapf	in vivo (rat)	Rats were either treated with 1 g/kg po β-myrcene in corn oil (n=unknown) or corn oil alone (n=unknown) 1 hour before a series of neurobehavioral tests were administrated.	• β-Myrcene had no protective effect on pentylenetetrazol (PTZ)-induced seizures in mice.<br>• β-Myrcene has no benzodiazepine-like anxiolytic activity.<br>• Activity on the central nervous system (antidepressive or antipsychotic) is unlikely	(da-Silva et al., 1991)
Anaesthetic	Synthetic β-myrcene (**purity unknown**)	in vivo (fish)	Experiment 1:<br>240 rainbow trout were treated to one concentration of either eugenol (12, 20, 30, 50, 80, and 130 μL/L) or myrcene (100, 150, 200, 300, 400, and 500 μL/L) concentrations. Induction time of and recovery time from anesthesia was recorded for each fish.	• β-Myrcene anesthetized trout within 60– 600 s at concentrations of 531–111 μL/L, which was markedly higher than the eugenol (81–10 μL/L).	(Mirghaed et al., 2018)

			Experiment 2:<br>48 rainbow trout were exposed to the calculated eugenol or β-myrcene concentrations. Blood samples were taken after the fish reached anaesthesia.		
Anaesthetic	Synthetic β-myrcene (**purity unknown**)	In vivo (Frog)	Frog (Rana nigromaculata) sciatic nerves were placed in ringer solution.<br><br>β-myrcene was first dissolved in dimethyl sulfoxide (DMSO) and then diluted to the final concentration in Ringer solution, where the concentration of DMSO was less than 1%.<br><br>Compound action potentials (CAPs) were recorded from the frog sciatic nerve by using the air-gap method.	• At 5 mmol/L of β-myrcene, the maximal concentration examined, there was minimal inhibition of CAPs.<br>• β-myrcene reduced CAP amplitudes by 7%, this was much lower compared to the other aroma oil components.	(Ohtsubo et al., 2015)

Sedative	Synthetic β-myrcene (**purity unknown**)	In vivo (mice)	The effects of β-myrcene (1.2 mM) on the $GABA_A$ receptor response were examined by using Xenopus oocyte expression system and an electrophysiological method.	• β-myrcene in beer caused little effect on the $GABA_A$ receptor response.<br>• Myrcenol which is produced from myrcene during boiling wort with hops, potentiated the $GABA_A$ receptor response significantly.	(Aoshima et al., 2006)
Sedative	Synthetic β-myrcene (**purity unknown)**	In vivo (rat)	Experiment 1:<br>A single dose of β-myrcene (0.25, 0.5 or 1.0 g/kg po) was given 1 h before pentobarbital (40 mg/kg ip).<br><br>Experiment 2:<br>Male rats were treated with β-myrcene (1.0 g/kg po once a day) for 14 days and injected with pentobarbital (40 mg/kg ip) 24 h after the last dose of β-myrcene.	• No effect was observed with the two lowest doses of β-myrcene.<br>• The highest β-myrcene dose given 1 h before pentobarbital increased the pentobarbital - induced sleeping time (131 +/- 15 min vs 64 +/- 15 min for controls, mean +/- SD).<br>• Repeated treatment with β-myrcene reduced pentobarbital sleeping time compared to the vehicle-treated control group (21 +/- 13 min vs 35 +/- 19 min for controls, mean +/- SD).<br>• β-myrcene induces the phenobarbital-inducible cytochrome P-450 (P-450 2B subfamily) enzymes in the rat.	(Freitas et al., 1993)
Antioxidant	Mt. Atlas mastic tree (**7.36% β-myrcene**); *Pistacia atlantica* Desf.	In vivo (rat)	50 adult male Wistar Rats were put into 4 groups (Each group had 6 rats):<br>(1) control group<br>(2) diabetic control group<br>(3) glibenclamide control group	Treatment with *Pistacia atlantica*:<br>• ↑ GSH, GPx, CAT and SOD levels<br>• ↓ MDA levels.	(Bagheri et al., 2019)

			(4) diabetic treated group with 200 mg/kg Pistacia atlantica oleoresin<br><br>Oxidative stress markers and antioxidant enzyme expression were investigated including: MDA< GSH, GPx and CAT.		
Antioxidant	Synthetic β-myrcene (**purity unknown)**	In vivo (fish)	Common carp (Cyprinus carpio) were fed with either myrcene-supplemented diets for 30 days before exposure to 0.5 mg/L unionized ammonia for 24 hours. The experimental diets contained 0, 0.1, 0.25, 0.5 and 1% of myrcene.<br><br>After 30 days growth performance was measured. Also, fish blood samples were analysed prior to and after the ammonia challenge.	• β-myrcene supplementation led to significant decrease in ALT, AST, ALP and LDH activities compared to the control group<br>• Significant antioxidant effects of β-myrcene were present preventing ammonia-induced tissue damages.	(Hoseini et al., 2019)

Antioxidant	Mastic oil (**26.21% β- myrcene**); *Pistacia lentiscus* L.	In vitro	Malondialdehyde (MDA), protein carbonyl (PC), and reduced glutathione (GSH) levels were examined in the ovaries and thyroid glands of Wistar rats.<br><br>Rats were put into 4 groups (Each group had 6 rats):<br>1. Rats were administered corn oil (4mL/kg body weight)<br>2. Rats were administered the *Pistacia lentiscus* oil (2mL/kg body weight).<br>3. Rats were given CPF in corn oil at a dose of 6.75mg/ kg body weight.<br>4. Rats were given *Pistacia lentiscus* oil (2mL/kg body weight) and after 2h, subjected to CPF treatment	Co-administration of *Pistacia lentiscus* oil and chlorpyrifos reduced oxidative damage: PC levels were restored, MDA levels lowered and GSH levels increased.	(Chebab et al., 2017)

Antioxidant	Synthetic β-myrcene (**purity: analytical grade or the highest grade available**	In vivo (mice)	C57BL/J6 male mice were put into 4 groups (Each group had 10 rats):<br>(1) control<br>(2) global cerebral I/R<br>(3) β-myrcene (200 mg/kg β-myrcene dissolved in 0.1 % carboxymethyl cellulose for 10 days) following a medial incision<br>(4) β-myrcene + I/R.	Treatment with β-myrcene:<br>• Protective against oxidative effects of global cerebral ischemia/reperfusion (cerebral I/R) by increasing GSH, GPx, CAT and SOD.<br>• Decreased the formation of TBARS.<br>• Eliminated the degenerate changes in heart tissue associated with cerebral I/R)	(Burcu et al., 2016)
Antioxidant	Synthetic β-myrcene (**purity unknown)**	In vivo (rat)	Adult male Wistar rats induced with ulcers were administered β-myrcene orally (gavage).	Treatment with β-myrcene:<br>• Protects the gastric and duodenal mucosa against ulcers and activates antioxidants such as GSH, NO, SH, GPX and GR	(Bonamin et al., 2014)
Antioxidant	Synthetic β-myrcene (**purity: analytical grade or the highest grade available)**	In vivo (rat)	Rats were put into 4 groups (Each group had 10 rats):<br>(1) sham-operated<br>(2) global cerebral I/R<br>(3) β-myrcene (200 mg/kg β-myrcene dissolved in 0.1 % carboxymethyl cellulose for 10 days) following a medial incision<br>(4) β-myrcene + I/R.	Treatment with β-myrcene:<br>• Protective against oxidative effects of global cerebral ischemia/reperfusion (cerebral I/R) by increasing GSH, GPx, and SOD.<br>• Decreased the formation of TBARS.<br>• Eliminated the neurodegenerative effects associated with cerebral I/R)	(Ciftci et al., 2014)

Antioxidant	Juniper berries EO (**8.3% β-myrcene**); *Juniperus communis* L., *Cupressaceae*	In vivo (*S. cerevisiae*)	• S. cerevisiae which was subjected to oxidative stress was placed in different concentrations of juniper berry oil (0.4, 0.8, 1.6, 3.2 and 4.0 mg/mL)<br>• The *S. cerevisiae* was evaluated in vivo on the antioxidant enzymes SOD, CAT and GPx	• The EO may block oxidation processes in yeast cells.<br>• The EO increased activity of antioxidant enzymes superoxide dismutase (SOD), catalase (CAT), and glutathione peroxidase (GPx).	(Höferl et al., 2014)
Antioxidant	Synthetic β-myrcene (**purity unknown)**	In vivo (rat)	Rats were put into 8 groups (Each group had 14 rats) who were administered the following orally by gavages:<br>(1) Negative control<br>(2) Positive control<br>(3) Curcumin<br>(4) β-myrcene at doses of 100 mg/kg/day, 200 mg/kg/day and 100 mg/kg/day, respectively.<br>(5) Cineole<br>(6) 2,3,7,8-tetrachlorodibenzo-	• Myrcene increased the level of GSH and the activities of SOD, CAT and GSH-Px in the liver<br>• Myrcene protects rat liver from oxidative damage induced by TCDD in a time- dependent manner.	(Ciftci et al., 2011a)

			p-dioxin (TCDD) + curcumin<br>(7) TCDD + β-myrcene<br>(8) TCDD + cineole<br><br>The liver samples were taken from half the rats (day 30) and the other half (day 60). The liver samples were used to determine TBARS, GSH, CAT, (GSH-Px)<br>and CuZn-SOD levels by spectrophotometric method.		
Anti-aging	Synthetic β-myrcene (**purity: analytical grade or the highest grade available)**	In vitro	UVB irradiated cultured human dermal fibroblasts from a skin biopsy of a healthy young male donor (South-Korea) were treated with 0.1, 1 and 10 μM of β-myrcene	Treatment with β-myrcene:<br>• ↓ ROS, MMP-1, MMP-3, and IL-6, and increased TGF 1 and type I procollagen secretions.<br>• ↓ The phosphorylation of various MAPK-related signalling molecules (p-ERK, p-p38, and p-JNK and AP-1 including p-c-Jun and p-c-Fos).	(Hwang et al., 2017)
Anti-inflammatory	*Santolina insularis* (Gennari ex Fiori) Arrigoni EO (**11.4% β-myrcene**)	In vitro	Mouse macrophages (RAW 264.7), were pre-treated with varying concentrations of the essential<br>oil (0.07 –1.05 mg/mL).	• The EO significantly reduced NO production without affecting macrophages viability.<br>• No scavenging NO scavenging potential was observed<br>• The essential oil inhibited the expression of two key pro-inflammatory enzymes, iNOS	(Alves-Silva et al., 2020)

			Anti-inflammatory activity of the EO was determined by:<br>• Measuring nitric oxide (NO) production<br>• Evaluating NO scavenging potential<br>• Measuring the expression of inducible nitric oxide synthase (iNOS) and cyclooxygenase-2 (COX-2).	and COX-2 (71% and 25% at 0.54 mg/mL).	
Anti-inflammatory	Synthetic β-myrcene (Purity unknown)	In vitro	Myrcene (50 mg/kg body weight, orally) was administered post Adrenalectomy to Male Wistar albino rats.	• β-myrcene resulted in the downregulation of pro-inflammatory cytokines and increase in anti-inflammatory cytokines.	(Islam et al., 2020)
Anti-inflammatory	Mastic gum oil (Mastic gum oil 1: **20.1%** and Mastic gum oil 2: **18.6%**); *Pistacia lentiscus* var. *chia*	In vitro	Mastic gums were collected from two wild trees around 100-years old (MGEO-1 and 2)<br><br>Lipopolysaccharide (LPS)-activated mouse macrophage RAW264.7 cells incubated were for 24 h with different MGEO concentrations (50, 5 or 0.5 µg/mL)	• In the iNOS assays, Mastic gum oil 1 and Mastic gum oil 2 were effective at inhibiting the LPS-stimulated production of NO, with inhibition following a dose-dependent response.	(Tabanca et al., 2020)

Anti-inflammatory	*Zanthoxylum leprieurii* Guill. & Perr. EO (**16.4 – 48.3% β-myrcene**)	In vitro	Anti-inflammatory activity of the EO was determined by:<br>• Inhibition Lipoxygenase Assay: Four dilutions of essential oils were prepared in methanol (25, 50, 75 and 100 µg/mL).<br>• Inhibition of Albumin Denaturation Assay (bovine serum albumin):<br>• Four dilutions of 1ml essential oil samples and diclofenac (standard) were prepared in methanol (25, 50, 75 and 100 µg/mL).	• The essential oils (leaves, trunk bark and fruit) showed high to moderate lipoxygenase inhibitory activity (IC_{50}: 26.26 – 32.42 µg/ml) in comparison to the standard Quercetin (21.57 µg/ml)<br>• In the anti-denaturation method of bovine serum, the essential oils (leaves, trunk bark and fruit) showed high to moderate anti-inflammatory activities (IC_{50}: 26.08 – 35.07 µg/ml) in comparison to the control Diclofenac (21.90 µg/ml)	(Tanoh et al., 2020)
Anti-inflammatory	Synthetic β-myrcene (**purity unknown)**	In vivo (rat)	Male Wistar rats were put into 4 groups (Each group had 2 rats) for 8 weeks:<br>(1) Control<br>(2) Isoproterenol (ISO)-induced heart failure	Treatment with β-myrcene:<br>• Reduced inflammatory cytokines, such as IL-6, IL-4, TNF-α, IFN-γ and IL-1β<br>• Improved IL-10 levels ($p < 0.01$), by blocking inflammatory signals in cardiac tissue.	(Tian et al., 2020)

			(3) β-Myrcene pre-treated (1.0 mg/kg/day and ISO)<br>(4) β-Myrcene was given as a drug control.<br><br>At the end of the treatment the rats were killed, and cardiac markers, anti-inflammatory and pro-inflammatory markers were analysed.		
Anti-inflammatory	*Santolina africana* Jord. & Fourr. (**4.2–20.9% β-myrcene**)	In vitro	Anti-inflammatory activity of the EO (1.5, 2.5, 5.0, 7.5 and 10.0 mg/mL) was determined by the lipoxygenase (LOX) inhibition activity assay.	• *S. africana* EO exhibits a high inhibition of lipoxygenases (LOX) activity, suggesting an anti-inflammatory potential.<br>• IC_{50} value (concentration at which 50% of the lipoxygenase was inhibited) of *Santolina africana* essential oil (0.065 ± 0.004 mg/mL) is 5-fold higher than IC_{50} value of nordihydroguaiaretic acid (NDGA) used as positive control.	(Malti et al., 2019)
Anti-inflammatory	*Limnocitrus littoralis* (Miq.) Swingle EO (**24.9 % β-myrcene**)	In vitro	Anti-inflammatory activity of the EO (100, 20, 4, and 0.8 µg/mL) was examined in LPS-stimulated RAW 264.7 cells by evaluating	• The essential oil of *L. littoralis* showed activity against the nitric oxide (NO) generation with the IC_{50} value to 12.50 ± 1.19 µg/L.	(Doan et al., 2019)

			the inhibition of Nitric oxide (NO) production.<br><br>L-N G - monomethyl arginine citrate (L-NMMA) was used as a positive control.		
Anti-inflammatory	*Santolina corsica* Jord. & Fourr. EO (**5.7 - 44.9% β-myrcene**)	In vitro	Samples of bronchial secrete coming from hospitalized patients suffering for respiratory diseases, were incubated with the essential oil (concentrations ranged between 0 and 1 µg/mL).	• *S. corsica* essential oil showed potential activity against respiratory infections with an inflammatory component.<br>• Incubation of *S. corsica* essential oil (1 µg/mL), had a positive impact on the decrease of granulocytes	(Foddai et al., 2019)
Anti-inflammatory	*Santolina corsica* Jord. & Fourr. (n-hexane extracts (EHS) (**18.86 % β-myrcene**)	In vitro	Lipopolysaccharide (LPS)-stimulated murine macrophages (RAW 264.7 cells) were treated for 24h with 0, 1, 6.25, 12.5 and 50µg/mL of EHS.<br><br>Anti-inflammatory activity was determined by using the Griess assay to measure NO production.	• EHS decreased NO production, showing anti-inflammatory activity.	(Bonesi et al., 2018)
Anti-inflammatory	• Synthetic β-myrcene **(≥95.0% β-myrcene**)<br><br>• *Eryngium duriaei* J.Gay ex Boiss subsp	In vitro	• Human chondrocytes of knee cartilage from the distal femoral condyles of multi-organ donors (20–70 years old, n=31) and patients (58–73 years old,	• NO and iNOS levels ↓ (Pro-inflammatory mediators)<br>• NF-κB ↓ (transcription factors)<br>• p38 and JNK activation ↓ (signal transduction).	(Rufino et al., 2015)

	juresianum (**trace amounts of β-myrcene**)		n=5) undergoing total knee arthroplasty.<br>• The human chondrocy-tic cell line, C28/I2, was used to evaluate NF-κB–DNA binding activity.<br>• The chondrocytes were treated with β-myrcene *(*ranging from 25 to 50 µg/ml) and were tested for the anti-inflammatory activities.		
Anti-inflammatory	Synthetic β-myrcene (**purity unknown**)	In vivo (rats)	β-Myrcene was administered orally (gavage) and dissolved in an 8% Tween-80 aqueous solution to adult male Wistar rats at doses of 3.75, 7.50 or 11.24 mg/kg bw.	• β-Myrcene at a dose of 7.50mg/kg has important anti-ulcer activity with significantly decreased gastric and duodenal lesions as well as increased gastric mucus production.<br>• ↑ enhancement of antioxidant enzyme activity from GR system<br>• ↑ glutathione peroxidase (GPx), glutathione reductase (GR), and total glutathione in gastric tissue.	(Bonamin et al., 2014)

Anti-inflammatory	*Cymbopogon citratus* (DC.) Stapf EO (**27.83 % β-myrcene**)	In vivo (mice)	Three-month old Wistar rats were orally fed with *C. citratus* essential oil at a dose ranging from 600 to 4,000 mg/kg.<br><br>Anti-inflammatory activity of the EO was determined on formol-induced edema in the animals.	After Formol-induced edema in the rats, *C. citratus* essential oil reduced the edema over time in a dose dependent manner.	(Gbenou et al., 2013)
Anti-inflammatory	Ginger EO (**14% β-myrcene**); *Zingiber officinale* Roscoe	In vivo (rat)	The essential oil (10^{-4}, 10^{-3} or 10^{-2} μg/ml), was administered orally to Male Wistar rats before a carrageenan injection (n=5).	• The number of leukocytes migrated to the perivascular tissue 4 h after the irritant stimulus diminished.<br>• The essential oil in all doses tested (10-4, 10 -3, or 10 -2 IL/mL) caused a significant reduction of leukocyte chemotaxis (35.89 ± 4.33, 30.67 ± 0.70, and 35.85 ± 3.83%, respectively) toward casein stimuli.	(Nogueira de Melo et al., 2011a)
Anti-inflammatory	Rosemary EO (**10.02% β-myrcene**); *Rosmarinus officinalis* L.	In vitro	The essential oil (125, 250, or 500mg/ kg), indomethacin (5mg/kg), or saline solution (sodium chloride 0.9%), was administered orally to Male Wistar rats before a carrageenan injection (n=5).	• The essential oil was involved in the inactivation of nuclear factor- κB, a transcription factor (regulating the transcription of many proinflammatory genes)<br>• The essential oil acted on cellular migration, reducing the in vitro leukocyte chemotaxis induced by casein for each concentration tested, contributing to its anti-inflammatory action.	(Nogueira de Melo et al., 2011b)

Anti-inflammatory	*Distichoselinum tenuifolium* (Lag.) F.García Mart. & Silvestre (7.7–84.6% **β-myrcene**)	In vitro	Mouse macrophages were in the presence of the essential oil at 0.64 µL/ml and 1.25 µL/ml.<br><br>Anti-inflammatory activity was determined using the Griess assay to measure NO production.	• The essential oil inhibited NO production induced by LPS in macrophages.	(Tavares et al., 2010)
Anti-inflammatory	*Eremanthus erythropappus* (DC.) MacLeish EO **(10.03% β-myrcene)**	in vivo (rat)	Carrageenan-induced oedema in rats and Carrageenan-induced pleurisy in rats:<br>• male Wistar rats (n=6 per test) were treated orally with essential oil (100, 200 and 400mgkg−1; 0.1 mL per 10 g body weight).	• Oral treatment with the essential oil of *E. erythropappus* markedly inhibited carrageenan-induced paw oedema in rats.<br>• Doses of 200 and 400 mg kg$(^{-1})$ administered 4 h before intrapleural injection of carrageenan significantly reduced exudate volume (by 20.20% and 48.70%, respectively) and leucocyte mobilization (by 5.88% and 17.29%, respectively).	(Sousa et al., 2008)
Anti-inflammatory	The essential oil from two Asteraceae species was analysed:<br>• *Porophyllum ruderale* (PR) (**16% β-myrcene**)<br>• *Conyza bonariensis* (CB) (**0.96% β-myrcene**)	In vitro	Peritoneal cells recovered from Balb/c mice were treated with β-myrcene (12.5, 25, 50 100, 200 µg/well)	• The essential oils, inhibited the LPS induced inflammation including cell migration<br>• Pure β-myrcene inhibited the production of NO at doses below the cytotoxicity of β-myrcene.<br>• A significant inhibition of γ-interferon and IL-4 production by β-myrcene was also observed.	(Souza et al., 2003)

	β-myrcene (**purity unknown**) was obtained from the essential oils.				
Anti-inflammatory	*Magnolia sieboldii* K.Koch EO (**12.72 % β-myrcene**)	In vitro	The effects of the essential oil (30 μg/ml), and β-myrcene (15 μM, 30 μM, 30μM) were examined on lipopolysaccharide (LPS)-induced production of nitric oxide (NO) and prostaglandin E_2 PGE_2 by rat peritoneal macrophages.	• The essential oil inhibited NO and PGE_2 production in a dose dependent manner.<br>• β-myrcene showed some inhibitory activity on NO and PGE_2 production by LPS-stimulated rat peritoneal macrophages, when assayed at a dosage 3x higher to what is usually present in the essential oil.	(Lim et al., 2002)
Analgesic	Synthetic β-myrcene (**purity unknown**)	In vitro	β-myrcene (10 to 150 μM) was applied to HEK TRexTRPV1 (rat) cell culture. β-myrcene was tested on its ability to induce Calcium influxes into a heterologous system with isolated expression of TRPV1.	• β-myrcene elicited large calcium influxes in a TRPV1-expression system and were blocked effectively by the TRPV1 antagonist Capsazepine.<br>• There is suggestive therapeutic potential of analgesic formulations containing β-myrcene.	(Jansen et al., 2019)
Analgesic	• *Ocimum gratissimum* L. EO (OgEO) (**0.24% β-myrcene**)	Randomized control trial	5 Male C57BL/6 J mice (3 months old) were administered drugs which were freshly diluted in corn oil on the day of each experiment and	• Mice treated with OgEO and their isolated active components (β-myrcene and eugenol) showed oral antinoceptive properties in mice	(Paula-Freire et al., 2016)

	• Synthetic β-myrcene (**purity is 95%**)		administered orally (p. o.) in a volume of 0.1 mL/10 g body weight,<br>always at 10: 00 a.m.<br><br>Mice were either treated with:<br>• corn oil (control and sham-operated)<br>• pregabalin (20 mg/kg, positive control, p. o.)<br>• Ocimum gratissimum essential oil (10, 20, or 40 mg/kg, p. o.)<br>• eugenol or β-myrcene (1, 5, or 10 mg/kg, p. o.)	subjected to chronic constriction injury<br>• OgEO and their isolated active components (β-myrcene and eugenol) have marked decrease in both thermal and mechanical hypernociception.<br>• The antinociceptive effect of β-myrcene seem to be mediated by the release of endogenous opioid-induced activation of α2 adrenergic receptors.	
Analgesic	• *Cymbopogon citratus* (DC.) Stapf EO (**27.83 % β-myrcene**)	In vivo (mice)	Three-month old Wistar rats were orally fed with C. citratus (n=6) essential oil at a dose ranging from 600 to 4,000 mg/kg.<br><br>Analgesic activity was determined by the tail immersion test.	• In the tail-immersion test, animals treated with 3000 mg/kg of C. citratus (8.44 ± 2.22 s) were able to keep their tails longer in a hot water bath (50°C) compared to untreated animals (4.75 ± 0.96 s), indicating its analgesic activity.	(Gbenou et al., 2013)

Analgesic	• *Ocimum gratissimum* L. EO (OgEO) (**0.24% β-myrcene**)<br>• Synthetic β-myrcene (**purity unknown)**	In vivo (mice)	Groups of 5 Adult male C57BL/6 J mice acutely received either:<br>• corn oil (control group, p.o.)<br>• OgEO (10, 20, or 40 mg/kg, p.o.)<br>• eugenol or β-myrcene (both at 1, 5, or 10 mg/kg, p.o.).<br>• One group received morphine (positive control group, 5 mg/kg, i.p.)<br>Antinociceptive activity was determined by the hot plate test and formalin test	• The highest doses of OgEO and β-myrcene significantly increased the latency to lick the paw(s) in the hot plate test compared with the control group.<br>• OgEO EO (40 mg/kg) and β-myrcene (10 mg/kg) was effective in minimizing animal pain in the first and second phases of the formalin test<br>• The antinociceptive effect shown by all drugs tested in hot plate test was reverted by naloxone administration (1 mg/kg), indicating opioid system participation.	(Paula-Freire et al., 2013)
Analgesic	*Teucrium stocksianum* Boiss. EO (**8.64 % Myrcene and 1.64% β-Myrcene**)	In vivo (mice)	Swiss Albino mice were split into groups receiving:<br>• 2.5% Tween-80 solution (10 ml/kg) was administered intraperitoneally to a control group (n=6)<br>• Doses of 20-160 mg/kg of essential oil (n=24)<br>• A standard group (n=6) was given an intraperitoneal	• There was an increase in percent writhe inhibition (PWI), which occurred from an essential oil dose of 20-80 mg/kg (b.w).<br>The maximum writhe inhibition was at 80 mg/kg (b.w) of essential oil, but PWI decreased at 160 mg/kg, which may be due to some toxic effect of higher dose.	(Shah et al., 2012)

			injection of 50 mg/kg dose of Diclofenic sodium.<br><br>Antinociceptive activity was determined by the acetic acid induced writhing method.		
Analgesic	*Eremanthus erythropappus* (DC.) MacLeish EO (**10.03% β-myrcene**)	In vivo (mice)	Acetic acid-induced writhing response in mice, Formalin-induced nociception in mice and Hot-plate latency assay in mice was assessed.<br><br>Male Swiss albino mice (n=8 per test) were treated orally with essential oil (100, 200 or 400 mgkg−1 ; 0.1 mL per 10 g body weight).	• The essential oil inhibited abdominal writhing induced by acetic acid (400 mgkg^{-1}) in mice by 27.06% compared with controls.<br>• In the formalin-induced nociception test in mice, the essential oil inhibited the first phase of paw licking by 29.13% (400 mgkg−1) and the second phase by 32.74% (200 mgkg−1) and 37.55% (400 mgkg−1).<br>In the hot-plate test in mice, doses of 200 mgkg−1 and 400 mgkg−1 significantly increased the reaction time after 30, 60 and 90 min of treatment, namely paw licking and jumping..	(Sousa et al., 2008)
Analgesic	Synthetic β-myrcene (**purity unknown)**	In vivo (rats)	A modification of the Randall-Selitto test was conducted:<br><br>3 paws of Male Wistar rats underwent intraplantar injections of increasing doses of β-myrcene (0-45 mg/kg/p.o.). The paws were subjected to the hyperalgesic effect of PGE_2.	The analgesic effects of β-myrcene are mediated by the arginine-NO-cGMP pathway.	(Duarte et al., 1992)

Analgesic	• Infusion of lemongrass (*Cymbopogon citratus* (DC.) Stapf) fresh leaves (**15-20% β-myrcene)**<br>• Synthetic β-myrcene (**purity of 95%)**	In vivo (rats)	Rat paw hyperalgesia test:<br>• oral administration of either a suspension of the lemongrass oil (0-120 mg/kg, n=15) or synthetic β-myrcene (0-135 mg/kg) 30 min before the different nociceptive stimuli to Wistar rats (n=5 per group)<br><br>Mouse writhing test:<br>• oral administration of either a suspension of the lemongrass oil (0- 405 mg/kg; n=10) or synthetic β-myrcene (0-405 mg/kg; n=10) 30 min before the different nociceptive injection to Swiss stock mice (n=5 per group)	• Silica gel column fractionation of the essential oil showed that β-myrcene was the major analgesic component in the oil (88%).<br>• Oral administration of an infusion of lemongrass fresh leaves produced a dose-dependent analgesic effect on rat paw hyperalgesia induced by isoprenaline and prostaglandin E2, but did not affect the one induced by DbcAMP (Similar results were seen in commercially administered β-myrcene).<br>There was a dose-dependent antinociception of the essential oil on both acetic acid- and iloprost-induced writhing in mice administered either a suspension of the lemongrass oil or β-myrcene.	(Lorenzetti et al., 1991)
Analgesic	*Cymbopogon citratus* (DC.) Stapf EO (**16% myrcene**)	In vivo (mice)	Hot plate test:<br>β-myrcene was administered at doses of 10 and 20 mg kg^{-1} (i.p.) (n=10)<br><br>Acetic acid test:<br>β-myrcene was administered at 20 and 40 mg kg^{-1} (s.c.), respectively (n=6 or 8)	• β-Myrcene acts at both central and peripheral sites as evidenced by an increase in reaction time of mice to thermal stimuli in the hot plate test and the decrease in the number of writhes to chemical stimuli in the acetic acid test.<br>• β-Myrcene induced analgesia was reversed by pre-treatment with naloxone in both tests suggesting	(Rao et al., 1990)

				the mediation of endogenous opioids in its mechanism.	

Supplementary table 5: Assessment of the potential toxicity of β-myrcene

Concentration of β -myrcene	Study population	Methodology	Findings	Reference
0.25 µg/mL, 0.50 µg/mL, and 1.0 µg/mL	Human lung cancer cell line (A549)	• Cytotoxicity was measured using the MTT (3-(4,5-Dimethylthiazol-2-yl)-2,5-diphenyltetrazolium bromide) assay.<br>• The ability of the cells to form colonies was tested using the Clonogenic Assay Kit.<br>• The mechanism of cell death was established using tetramethylrhodamine ethyl ester (TMRE)-mitochondrial membrane potential Assay Kit.<br>• The activity of caspase-3 (b39401) and caspase-9 was measured using commercial assay kits.	• β-Myrcene is involved in the antiproliferation and apoptosis of A549 cells.<br>• β-Myrcene induced apoptosis in a dose-dependent manner while inducing reactive oxygen species levels.<br>• In β-myrcene treated A549 cells, Caspase-3 activities increased with reduced mitochondrial membrane potential synthesis, suggesting the possibility of A549 cells being toxic to β-myrcene.	(Bai and Tang, 2020)
2.5, 5, 10, 25, 50, 100, 250, 500 or 1000 µg/ml	• Human peripheral blood mononuclear cells (PBMC) (non-metabolizing cells), were isolated from two male and two female non-smoking donors.	Cytotoxicity was assessed in human cells using the MTT assay. Genotoxicity was assessed using the comet assay.	• β-Myrcene significantly reduced leukocyte cell viability at concentrations above 100 µg/ml.<br>• After metabolic activation by HepG2/C3A cells, β-myrcene did not produce	(Orlando et al., 2019)

	• Human hepatoma cell line (HepG2/C3A) (metabolizing cells)		marked change in cell viability.<br>• In the MTT assay, β-myrcene showed cytotoxicity above 250 µg/ml in human leukocytes, compared to human HepG2/C3A liver cells (no cytotoxicity observed).<br>• β-Myrcene at concentrations of 100 and 1000 µg/ml demonstrated significant DNA damage, when assessed using the comet assay.	
Diets containing 0, 50, 150, or 300 mg/kg bw/day of myrcene (purity of 93.3%) were administered in a 90-day period.	Sprague Dawley rats (10/sex/group)	After rats consumed β-myrcene, clinical observations, hematology, clinical chemistry parameters, organ weights, macroscopic and histopathological examinations were made.	• This study shows no effects attributable to the ingestion of β-myrcene on clinical observations, hematology and kidney weights.<br>• At the highest dose tested, there were no observed adverse effects.	(Bastaki et al., 2018)
0.03, 0.1 or 0.3 µl/plate	TA98 and TA1537 tester strains of Salmonella typhimurium	β-Myrcene was tested individually and in combination with promutagens 2-aminoanthracene (2AA) and benzo(a)pyrene (BP) in presence of metabolic activation system (Rat liver S9) by plate incorporation method.	• β-Myrcene enhanced the mutagenic responses of promutagens (2AA and BP) under metabolically activated conditions.<br>• In-silico analysis predicted a mutagenic alert for β-myrcene, due to the presence of conjugated alkene alerts.	(Kanode et al., 2017)

		The chemical structure of β-myrcene was analysed by *Derek-Nexus*.		
0.8 mg/ml, i.p., for 6 weeks given at 3-week intervals (starting at the same time of immunization)	Groups of 6 BALB/c mice	Mice were immunized with β-myrcene mixed with Ovalbumin (OVA) or Ag85B (a protective antigen for tuberculosis)	• β-Myrcene enhanced specific antibody responses against OVA and Ag85B.<br>• β-Myrcene increased IgG titers in immunized mice<br>• When β-myrcene was administered alone it did not enhance levels of T-helper Th1 and Th2 cytokines or increase immunoglobulin IgG subtypes.	(Uyeda et al., 2016)
50 or 100 µM β-myrcene	Metastatic MDA-MB-231 human breast cancer cells	The following assays were conducted;<br>• MMP-9 promoter reporter assay<br>• Cytotoxicity assay<br>• NF-κB-dependent transcriptional activity assay<br>• Reverse-transcription polymerase chain reaction and quantitative real-time PCR<br>• Immunoblot analysis<br>• Immunofluorescence staining	β-Myrcene inhibits tumor necrosis factor-α (TNFα)-induced nuclear factor κB (NF-κB) activity through suppression of κB kinase and downregulation of matrix metalloproteinase-9. Subsequently, anti-metastatic activity of breast cells is promoted.	(Lee et al., 2015)

		• Three-dimensional spheroid cell invasion assay		
90-day Toxicity Study:<br>0, 250, 500, 1,000, 2,000, and 4,000 mg/kg body weight (> 90% purity)<br><br>2 year toxicity study:<br>0, 250, 500, and 1,000 mg/kg body weight (> 93% purity)	90-day Toxicity Study:<br>Groups of 10 male and 10 female F344/N rats<br><br>2 year toxicity study:<br>Groups of 50 male and 50 female F344/N rats	90-day Toxicity Study:<br>Rats were administered β-myrcene in corn oil by gavage, 5 days per week for 14 weeks. All rats underwent complete necropsies and microscopic examinations.<br>2 year toxicity study:<br>Rats were administered β-myrcene in corn oil by gavage, 5 days per week for 105 weeks. All rats underwent complete necropsies and microscopic examinations.	β-Myrcene produced α2u-g nephropathy at the lower doses in the 90-day study and linear papillary mineralization at lower doses in the 2-year study in males.<br><br>Nephrosis (characterised by dilation of the S3 tubules, nuclear enlargement and luminal pyknotic cells of the outer stripe of the outer medulla) was minimal at higher doses in the 90 day study. On the other hand, nephrosis showed a direct dose-correlation in males in the 2 year study.<br>Renal tubule tumours were more prominent in males treated with β-myrcene at low dosages (250 and 500 mg/kg), where there was an incidence of up to 30%. All treated groups had a low incidence of tumours, except for the control males and females.	(Cesta et al., 2013)
Concentration of β-myrcene is not known but purity was ≥90%	HepG2 (hepatocellular carcinomic human cell line), B16F10 (murine melanoma), K562 (erythromyeloblastoid leukemia cell line)	Cytotoxicity was tested by using the methyl-[3H]-thymidine incorporation assay.	β-Myrcene displayed cytotoxicity to different tumour cell lines, the IC50 values are as followed:<br>• HepG2 =9.23 µg/ml<br>• B16F10 =12.27 µg/ml<br>• K562 = Not determined	(Ferraz et al., 2013)

0.39– 200 μ g/ml of *Vepris macrophylla* (Baker) I.Verd (8.3% of β-myrcene).	Human glioblastoma multiformecell line, Human breast adenocarcinoma cell, Human malignant melanoma cell and Human colon carcinoma cell line.	Cytotoxicity was measured using the MTT assay.	*Vepris macrophylla* was cytotoxic against MDA-MB 231 (human breast adenocarcinoma) and HCT116 (human colon carcinoma) cell lines. The inhibitory effects were comparable with cisplatin.	(Maggi et al., 2013)
200 mg/kg bw per day, for 30 or 60 days	Young adult female Wistar Albino rats	β-Myrcene orally administered by gavages dissolved in corn oil with and without 2,3,7,8-tetrachlorodibenzo-p-dioxin (TCDD, 2 μg/kg bw per week by gavage); an environmental pollutant. Blood samples were stored for flow cytometric analysis.	• β-Myrcene reduced the percentage of CD8+ cells in the blood, while increasing the percentages of CD3+, CD4+, CD161+, CD45RA, CD4+CD25+, and the populations of total lymphocyte cells .<br>• β-Myrcene showed immunomodulatory effects and counteracted the immunosuppressive effects induced by TCDD (when administered concomitantly).	(Ciftci et al., 2011b)
0, 0.25, 0.5, 1 g/kg of bw	Male and Female F344/N Rats; Male and Female B6C3F1 Mice	β-Myrcene in corn oil was given by gavage directly into the stomach to 50 male and females for three months or two years. Control animals used corn oil without any β-myrcene using the same method. At the end of the study, 40 sites were examined in every animal.	• All male rats receiving 1.0 g/kg of β-myrcene died before the end of the study (100% fatality)<br>• Most male (58% fatality) and female (66% fatality) mice receiving 1.0 g/kg died before the end of the study<br>• The incidence of renal tumours increased in male rats receiving doses of 0.25 and 0.5 g/kg of β-myrcene. Some female	(NTP, 2010)

			rats also developed renal tumours, to a lesser extent.<br>• Male mice had an increase in the development of adenomas and carcinomas of the liver. Similarly, females developed liver tumours to a lesser extent.	
33 -10000 µg/plate	*Salmonella typhimurium* (TA97, TA98, TA100, and TA1535) or *Escherichia coli strain* WP2 uvrA pKM101	Mutagenicity was assessed using the Ames test	• No mutagenic activity of β-myrcene was observed in any of the bacterial strains in the presence or in the absence of exogenous metabolic activation (S9 fraction from Aroclor 1254-induced rat or hamster liver).	(NTP, 2010)
0.25-2 g/kg	Mouse, b6c3f1 (m and f)	Mice were administered β-myrcene for 3 months by gavage. After 3 months, peripheral blood samples were obtained from mice and the peripheral blood micronucleus assay was carried out.	• No significant increases in the frequencies of micronucleated normochromatic erythrocytes was observed<br>• β-Myrcene was not display bone marrow toxicity, given the increase in the percentage of reticulocytes among total erythrocytes.	(NTP, 2010)
Not known	human breast cancer (MCF-7) and normal Chang liver cell lines.	Cytotoxicity was measured using the MTT assay.	• β-Myrcene inhibited proliferation of MCF-7 cells in vitro, with an IC50 of 291 µM .<br>• β-Myrcene was mildly toxic to normal Chang liver cells, with an IC50 of 9.5 mM.	(Chaouki et al., 2009)

Bacteria: 0.05–1.5 mg/plate<br><br>Mammalian cells: 0.01, 0.1, 1 and 10 µg/ml	• Escherichia coli WP2 IC185 and oxyR mutant IC202<br>• hepatoma HepG2 and human B lymphoid NC–NC cells	• The Reverse mutation assay was used to evaluate the mutagenic and antimutagenic potential of β-myrcene with Escherichia coli WP2 IC185 and its oxyR mutant IC202 strain<br>• Cytotoxicity was measured using the MTT assay.<br>• Genotoxicity and antigenotoxicity of monoterpenes were evaluated using the comet assay.	• β-Myrcene reduced the number of t-BOOH induced revertants by 42.6–80.2% in the bacterial assay.<br>• β-Myrcene reduced t-BOOH induced DNA damage by approximately 50% at 1.0 µg/ml in NC-NC cells.<br>• In HepG2 cells, β-myrcene was ineffective at reducing tert-butyl hydroperoxide-induced DNA damage	(Mitić-Ćulafić et al., 2009)
0.5, 2.5 or 5mg/paw	26 Wistar female rats	The rat popliteal lymph node assay (PLNA), was used as a screening test for allergic and autoimmune-like reactions. Rats were injected with 50 microL of β-myrcene into the right hind foodpad, while the contralateral left hind footpad was injected with the vehicle (DMSO).	• In the primary (direct) PLNA, β-myrcene produced a clear immuno-stimulatory response due to its irritant properties.<br>• In the secondary PLNA (T cell priming test), β-myrcene was not an immune-sensitizing agent.	(Friedrich et al., 2007)
>200 µg/mL	HeLa (human cervical carcinoma), A-549 (human lung carcinoma), HT-29 (human colon adenocarcinoma) cell lines	Cytotoxicity was measured using the MTT assay	• β-Myrcene over a concentration of 200 µg/ml is cytotoxic against HeLa, A-549, HT-29 cell lines.	(Silva et al., 2007)

Without metabolic activation: 10 - 5000 µg/plate<br><br>With metabolic activation: 1 - 1500 µg/plate	*Salmonella typhimurium*, TA97a, TA98, TA100, TA1535	Mutagenicity of β-myrcene was evaluated by the Salmonella/microsome assay (Ames test)	• β-myrcene is not mutagenic in the Ames test.	(Gomes-Carneiro et al., 2005)
3.0% (w/w) oxidized β-myrcene (synthetic), containing 30% β -myrcene	1511 consecutive dermatitis patients (humans) in 6 European dermatology centres (Copenhagen, Dortmund, Leuven, London, Malmo¨ and Odense)	Participants were patch tested with oxidized fragrance terpenes and some oxidation fractions and compounds.	0.07% of patients reacted adversely to β-myrcene.	(Matura et al., 2005)
0.05 ml	11 Guinea pigs	Tea-tree oil sensitive guinea pigs were patch tested with β-myrcene on the clipped and shaved right flank of the animals.	Two guinea pigs reacted adversely to β-myrcene	(Hausen et al., 1999)
10 µL	Not applicable	β-myrcene was studied for inhibitory effects on the formation of N-nitrosodimethylamine (NDMA).<br>The reaction mixture consisted of dimethylamine and sodium nitrite adjusted at pH 3.6, in addition to β-myrcene and an emulsifying agent.	β-Myrcene inhibited the in vitro formation of NDMA, a potent carcinogen in experimental animals, by 88%.	(Sawamura et al., 1999)

100, 300 and 500 mg/kg body weight	Male (n=15 per dose group) and female Wistar rats (n=45 per dose group)	β-Myrcene was administered by gavage dissolved in peanut oil to male rats for 91 days prior to mating and during the mating period. β-myrcene was given to females prior to mating, during mating, pregnancy, and during lactation until day 21 after parturition. All rats were evaluated for development, mortality, and signs of toxicity. Reproductive and foetal developmental abnormalities in Wistar rats was also evaluated.	• The NOAEL for toxic effects on fertility and general reproductive performance was 300 mg for β-myrcene/kg body weight, when administered orally.<br>• β-myrcene increased liver and kidney weights, but no other toxicity was observed in the rats.<br>• β-Myrcene did not affect the mating index or pregnancy index.<br>• β-Myrcene did not cause maternal toxicity or externally visible malformations. However, at the highest dose (500 mg/kg), there was a higher frequency of skeletal anomalies.<br>• There was no effects on labour or pup mortality/<br>• There was no adverse effects on postnatal weight gain or offspring body weight during lactation.<br>• There was a minor effect on postnatal development on offspring (delay in eye-opening, coat development and incisor eruption).	(Paumgartten et al., 1998)

<10–2 mug/mL	Crown gall tumors, MCF-7 breast carcinoma, HT-29 colon	No methodology available	β-Myrcene showed significant cytotoxic effects in crown gall tumors (50% inhibition), MCF-7 breast carcinoma, HT-29 colon adenocarcinoma	(Saleh, 1998)
0.02–1 μM in 10 μl in dimethylsulfoxide (DMSO)	Liver microsomes from female Wistar rats	The inhibitory effects of β-myrcene was studied on liver monooxygenases involved in the activation of genotoxic substances.	β-Myrcene inhibited the activity of pentoxyresorufin-O-depenthylase (PROD), a selective marker for mono-oxygenase CYP2B1, necessary for the activation of genotoxins in rats.	(De-Oliveira et al., 1997)
0.25, 0.5 and 1.2 g/kg	29 Wistar female rats	β-Myrcene in corn oil was given orally from day 6 to 15 of pregnancy.<br>On day 20, caesarean sections were performed.<br>Foetuses were examined for skeletal, visceral and external malformations.	• No evidence that β-myrcene is a teratogenic substance.<br>• No adverse effects were seen with the two lowest doses tested.<br>• Highest dose of β-myrcene (1.2 g/kg) induced maternal toxicity by decreasing weight gain with a higher incidence of signs of retardation and of anomalies in the foetal skeleton	(Delgado et al., 1993a)
0.25, 0.5, 1.0 and 1.5 g/kg	Female wistar rats	Doses of myrcene were administered by gavage from day 15 of pregnancy, parturition and throughout the period of lactation up to weaning (postnatal day 21).<br>Mortality, weight gain, physical signs of postnatal	• No adverse effects for peri- and post- natal development at the lowest dose tested was observed (0.25 g myrcene/kg body weight)<br>• Doses as high as 1.0 and 1.5 g/kg, were shown to	(Delgado et al., 1993b)

		development and reproductive capacity were evaluated in offspring.	impair female offspring fertility and reduce birth weight.	
0.5 – 10 mg	*Salmonella typhimurium* strains TA98 and TA100	Mutagenicity of β-myrcene was evaluated by the Salmonella/microsome assay (Ames test)	• β−Myrcene exhibited antimutagenic properties in a dose-dependent manner towards chemical-induced mutation (AFB, Trp-P-1, Trp-P-2, Glu-P-1, Glu-P-2, IQ, MNNG and AF-2) in *S. typhimurium* strains.<br>• No effect of β-myrcene was observed on the mutagenic activity of<br>• benzo[α]pyrene.	(Vinitketkumnuen et al., 1994)
0.1, 0.5 and 1.0 g/kg po	Two or four Wistar rats of either sex.	β-Myrcene was administered orally by gavage once. Bone marrow cells were harvested 24 and 48 h after β-myrcene administration.<br>The mitotic index and the frequency of chromosome aberrations were evaluated.	• No evidence of the genotoxicity of β-myrcene is present.<br>• In vitro mutagenicity tests were negative and there was no evidence of β-myrcene -induced clastogenicity was observed<br>• 24 hours after β-myrcene, a dose related increase in mitotic index was observed.	(Zamith et al., 1993)

Not determined	Mouse P388 leukemia cell	No methodology available	• Significant cytotoxicity was observed against P388 leukemia cell.	(Okamura et al., 1993)
1.5-3.0% in DMSO	Salmonella typhimurium (TA100)	Mutagenicity of β-myrcene was evaluated by the Salmonella/microsome assay (Ames test)	• Doses of 1.5 and 3.0% of β−Myrcene, showed inhibitory actions of 65 and 73% respectively when tested with 1.0 µg/plate AFB1 in TA100	(Kim, 1992)
Up to 1000 µg/mL	V79 cell line (Chinese hamster, lung)	The genotoxicity of β-myrcene was evaluated in mammalian cells in vitro using the V79 HPRT gene mutation test. Myrcene was tested in the presence and absence of S9-mix.	• Myrcene did not cause increased mutation frequencies at the hprt-locus in V79-cells.<br>• Tests with and without S9-mix revealed negative results.	(Kauderer et al., 1991)
100, 500, or 1000 µg/mL	Lymphocytes were isolated from one male and one female non-smoking donors.	Lymphocytes treated with β-myrcene were analysed for Chromosomal aberrations and sister-chromatid exchanges.	• β-Myrcene did not induce chromosome aberrations or sister-chromatid exchange when tested with human lymphocytes.<br>• The mitotic index nor the proliferation index was influenced by β-myrcene treatment.<br>• β-Myrcene has antimutagenic effects.	(Kauderer et al., 1991)

100- 500 µg/mL	V79 cell line (Chinese hamster, lung)	The influence of β-myrcene on sister-chromatid exchanges in v79 cells induced by 4 S9 mix activated indirect mutagens was studied using the 'sister chromatid exchange' (SCE) assay<br><br>The 4 mutagens were: cyclophosphamide (CP), benzo[a]pyrene (BP), aflatoxin B1 (AFB) and 9,10-dimethyl-1,2-benz[a]amhracene (DMBA).	• β-Myrcene inhibited sister-chromatid exchanges induced by CP and AFB in a dose-dependent manner.<br>• No effect of β-myrcene on sister-chromatid exchanges induction by BP and DMBA was observed.<br>• β-Myrcene may inhibit certain forms of cytochrome P-450 enzymes, required for activating pre-mutagens such as CP and AFB.	(Röscheisen et al., 1991)
100- 500 µg/mL	HTC cell line (rat hepatocellular carcinoma)	The influence of β-myrcene on sister-chromatid exchanges in v79 cells induced by S9 mix activated indirect mutagens was studied using the 'sister chromatid exchange' (SCE) assay.	• β-Myrcene caused a slight increase of SCEs in HTC cells after treatment for 1 cell cycle (20 h) in the presence of bromodeoxyuridine.<br>• β-myrcene was shown to reduce CP-induced SCE frequencies in a hepatic tumor cell line (HTC).	(Röscheisen et al., 1991)
Article not available	Rats and Mice	Article not available	• Necropsy data revealed no alterations in mice, upon β-myrcene administration.<br>• Histopathology findings in rats, indicated that β-myrcene could potentially induce liver and stomach toxicity.	(Paumgartten et al., 1990)

			• The oral approximate lethal doses of β-myrcene in mice and rats were 5.06 g/kg and 11.39 g/kg, respectively.<br>• The intraperitoneal approximate lethal doses of β-myrcene in mice and rats were 2.25 g/kg and 5.06 g/kg, respectively.	
The experimental diets consisted of powdered Wayne Lab Blox with 1 % (w/w) of β-myrcene (Purity of 99.0%)	Female Sprague—Dawley rats (32 rats were fed a diet of β-myrcene)	Rats were chemically induced with tumours using the 'DMBA-induced mammary carcinogenesis' model. The time taken for the appearance of first tumour (tumor latency) was measured	• β-Myrcene did not extend mammary tumour latency and did not reduce the total number of mammary tumours in Sprague-Dawley rats, when compared to controls.	(Russin et al., 1989)
Article not available	Humans	Article not available	• Exposure of β-myrcene can cause dermatitis, conjunctivitis, somnolence, and asthma-like symptoms	(Newmark, 1978)
4% in petrolatum	Humans (n=25)	A maximization test was carried out for assessing skin sensitization potential	• β-Myrcene produced no sensitization reactions	(Kligman, 1972)

Undiluted β-myrcene	Rabbits and rats	• β-Myrcene was applied to abraded or intract skin for 24 hours under occlusion.<br>• The median lethal dose (or LD50) was calculated for rabbits and rats.	• β-Myrcene was moderately irritating to rabbits.<br>• The acute oral LD_{50} value in rats and acute dermal LD_{50} value in rabbits exceeded 5g/kg.	(Moreno, 1972)

Aberl, A., and Coelhan, M. (2012). Determination of volatile compounds in different hop varieties by headspace-trap GC/MS--in comparison with conventional hop essential oil analysis. *J Agric Food Chem* 60(11)**,** 2785-2792. doi: 10.1021/jf205002p.

Aćimović, M.G., Pavlović, S., Varga, A.O., Filipović, V.M., Cvetković, M.T., Stanković, J.M., et al. (2017). Chemical Composition and Antibacterial Activity of Angelica archangelica Root Essential Oil. *Nat Prod Commun* 12(2)**,** 205-206.

Aghaei, Y., Hossein Mirjalili, M., and Nazeri, V. (2013). Chemical Diversity among the Essential Oils of Wild Populations of Stachys lavandulifolia Vahl (Lamiaceae) from Iran. *Chemistry & Biodiversity* 10(2)**,** 262-273. doi: https://doi.org/10.1002/cbdv.201200194.

Alitonou, G., Noudogbessi, J.-P., Philippe, S., Tonouhewa, A., Avlessi, F., Menut, C., et al. (2012). Chemical composition and biological activities of essential oils of Pimenta racemosa (Mill.) J. W. Moore. from Benin. *International Journal of Biosciences* 2**,** 1-12.

Alves-Silva, J.M., Piras, A., Porcedda, S., Falconieri, D., Maxia, A., Gonçalves, M.J., et al. (2020). Chemical characterization and bioactivity of the essential oil from Santolina insularis, a Sardinian endemism. *Nat Prod Res***,** 1-5. doi: 10.1080/14786419.2020.1774764.

Andrea, V., Nadia, N., Teresa, R.M., and Andrea, A. (2003). Analysis of Some Italian Lemon Liquors (Limoncello). *Journal of Agricultural and Food Chemistry* 51(17)**,** 4978-4983. doi: 10.1021/jf030083d.

Aoshima, H., Takeda, K., Okita, Y., Hossain, S.J., Koda, H., and Kiso, Y. (2006). Effects of beer and hop on ionotropic gamma-aminobutyric acid receptors. *J Agric Food Chem* 54(7)**,** 2514-2519. doi: 10.1021/jf051562a.

Ayedoun, A.M., Adeoti, B.S., Setondji, J., Menut, C., Lamaty, G., and Bessiére, J.-M. (1996). Aromatic Plants from Tropical West Africa. IV. Chemical Composition of Leaf Oil of Pimenta racemosa (Miller) J. W. Moore var. racemosa from Benin. *Journal of Essential Oil Research* 8(2)**,** 207-209. doi: 10.1080/10412905.1996.9700597.

Bagheri, S., Sarabi, M.M., Khosravi, P., Khorramabadi, R.M., Veiskarami, S., Ahmadvand, H., et al. (2019). Effects of Pistacia atlantica on Oxidative Stress Markers and Antioxidant Enzymes Expression in Diabetic Rats. *J Am Coll Nutr* 38(3)**,** 267-274. doi: 10.1080/07315724.2018.1482577.

Bai, X., and Tang, J. (2020). Myrcene Exhibits Antitumor Activity Against Lung Cancer Cells by Inducing Oxidative Stress and Apoptosis Mechanisms. *Natural Product Communications* 15(9)**,** 1934578X20961189. doi: 10.1177/1934578X20961189.

Baik, J.S., Kim, S.S., Lee, J.A., Oh, T.H., Kim, J.Y., Lee, N.H., et al. (2008). Chemical composition and biological activities of essential oils extracted from Korean endemic citrus species. *J Microbiol Biotechnol* 18(1)**,** 74-79.

Baser, K., Demirci, B., Ozek, T., Viljoen, A., and Victor, J. (2006). Composition of the essential oils of five Coleonema species from South Africa. *Journal of Essential Oil Research* 18(3)**,** 26-29.

Bassolé, I.H.N., Lamien-Meda, A., Bayala, B., Obame, L.C., Ilboudo, A.J., Franz, C., et al. (2011). Chemical composition and antimicrobial activity of Cymbopogon citratus and Cymbopogon giganteus essential oils alone and in combination. *Phytomedicine* 18(12)**,** 1070-1074. doi: https://doi.org/10.1016/j.phymed.2011.05.009.

Bastaki, M., Aubanel, M., Bauter, M., Cachet, T., Demyttenaere, J., Diop, M.M., et al. (2018). Absence of renal adverse effects from β-myrcene dietary administration in OECD guideline-compliant subchronic toxicity study. *Food and chemical toxicology : an international journal published for the British Industrial Biological Research Association* 120**,** 222-229. doi: 10.1016/j.fct.2018.07.004.

Bernard, C. (2001). Essential Oils of Three Angelica L. Species Growing in France. Part II: Fruit Oils. *Journal of Essential Oil Research - J ESSENT OIL RES* 13**,** 260-263. doi: 10.1080/10412905.2001.9699689.

Bonamin, F., Moraes, T.M., Dos Santos, R.C., Kushima, H., Faria, F.M., Silva, M.A., et al. (2014). The effect of a minor constituent of essential oil from Citrus aurantium: the role of β-myrcene in preventing peptic ulcer disease. *Chem Biol Interact* 212**,** 11-19. doi: 10.1016/j.cbi.2014.01.009.

Bonesi, M., Brindisi, M., Armentano, B., Curcio, R., Sicari, V., Loizzo, M.R., et al. (2018). Exploring the anti-proliferative, pro-apoptotic, and antioxidant properties of Santolina corsica Jord. & Fourr. (Asteraceae). *Biomed Pharmacother* 107**,** 967-978. doi: 10.1016/j.biopha.2018.08.090.

Bruns, K., and Meiertoberens, M. (1987). Volatile constituents of Pteronia incana (compositae). *Flavour and Fragrance Journal* 2(4)**,** 157-162. doi: https://doi.org/10.1002/ffj.2730020404.

Burcu, G.B., Osman, C., Aslı, C., Namik, O.M., and Neşe, B.T. (2016). The protective cardiac effects of Β-myrcene after global cerebral ıschemia/reperfusion in C57BL/J6 mouse. *Acta Cir Bras* 31(7)**,** 456-462. doi: 10.1590/s0102-865020160070000005.

Burdock, G.A. (2019). *Fenaroli's Handbook of Flavor Ingredients: Volume 2.* CRC press.

Butkienë, R., Nivinskienë, O., and Mockutë, D. (2004). Chemical composition of unripe and ripe berry essential oils of Juniperus communis L. growing wild in Vilnius district. *Chemija* 15(4)**,** 57-63.

Calín-Sánchez, Á., Martínez, J.J., Vázquez-Araújo, L., Burló, F., Melgarejo, P., and Carbonell-Barrachina, Á.A. (2011). Volatile composition and sensory quality of Spanish pomegranates (Punica granatum L.). *Journal of the Science of Food and Agriculture* 91(3)**,** 586-592. doi: 10.1002/jsfa.4230.

Câmara, C.C., Nascimento, N.R., Macêdo-Filho, C.L., Almeida, F.B., and Fonteles, M.C. (2003). Antispasmodic effect of the essential oil of Plectranthus barbatus and some major constituents on the guinea-pig ileum. *Planta Med* 69(12)**,** 1080-1085. doi: 10.1055/s-2003-45186.

Cesta, M.F., Hard, G.C., Boyce, J.T., Ryan, M.J., Chan, P.C., and Sills, R.C. (2013). Complex histopathologic response in rat kidney to oral β-myrcene: an unusual dose-related nephrosis and low-dose alpha2u-globulin nephropathy. *Toxicol Pathol* 41(8), 1068-1077. doi: 10.1177/0192623313482057.

Chagonda, L.S., Makanda, C., and Chalchat, J.-C. (2000a). Essential Oils of Cultivated Cymbopogon winterianus (Jowitt) and of C. citratus (DC) (Stapf) from Zimbabwe. *Journal of Essential Oil Research* 12(4), 478-480. doi: 10.1080/10412905.2000.9699570.

Chagonda, L.S., Makanda, C., and Chalchat, J.-C. (2000b). The essential oils of wild and cultivated Cymbopogon validus (Stapf) Stapf ex Burtt Davy and Elionurus muticus (Spreng.) Kunth from Zimbabwe. *Flavour and Fragrance Journal* 15(2), 100-104. doi: https://doi.org/10.1002/(SICI)1099-1026(200003/04)15:2<100::AID-FFJ874>3.0.CO;2-Y.

Chaouki, W., Leger, D.Y., Liagre, B., Beneytout, J.L., and Hmamouchi, M. (2009). Citral inhibits cell proliferation and induces apoptosis and cell cycle arrest in MCF-7 cells. *Fundam Clin Pharmacol* 23(5), 549-556. doi: 10.1111/j.1472-8206.2009.00738.x.

Chebab, S., Mekircha, F., and Leghouchi, E. (2017). Potential protective effect of Pistacia lentiscus oil against chlorpyrifos-induced hormonal changes and oxidative damage in ovaries and thyroid of female rats. *Biomed Pharmacother* 96, 1310-1316. doi: 10.1016/j.biopha.2017.11.081.

Chen, H.C., Tsaia, Y., Jr., Linb, L.Y., Wu, C.S., Tai, S.P., Chen, Y.C., et al. (2014). Volatile compounds from roots, stems and leaves of Angelica acutiloba growing in Taiwan. *Nat Prod Commun* 9(4), 583-586.

Ciftci, O., Ozdemir, I., Tanyildizi, S., Yildiz, S., and Oguzturk, H. (2011a). Antioxidative effects of curcumin, β-myrcene and 1,8-cineole against 2,3,7,8-tetrachlorodibenzo-p-dioxin-induced oxidative stress in rats liver. *Toxicol Ind Health* 27(5), 447-453. doi: 10.1177/0748233710388452.

Ciftci, O., Oztanir, M.N., and Cetin, A. (2014). Neuroprotective effects of β-myrcene following global cerebral ischemia/reperfusion-mediated oxidative and neuronal damage in a C57BL/J6 mouse. *Neurochem Res* 39(9), 1717-1723. doi: 10.1007/s11064-014-1365-4.

Ciftci, O., Tanyildizi, S., and Godekmerdan, A. (2011b). Curcumin, myrecen and cineol modulate the percentage of lymphocyte subsets altered by 2,3,7, 8-Tetracholorodibenzo-p-dioxins (TCDD) in rats. *Human & Experimental Toxicology* 30(12), 1986-1994. doi: 10.1177/0960327111404909.

Contreras, B., Rojas, J., Celis, M., Rojas, L., Mendez, L., and Landrum, L. (2014). Volatile compounds of Pimenta racemosa var. racemosa (Mill.) JW Moore (Myrtaceae) leaves from Tachira-Venezuela. *Boletin Latinoamericano y del Caribe de Plantas Medicinales y Aromaticas* 13, 305-310.

da-Silva, V.A., de-Freitas, J.C., Mattos, A.P., Paiva-Gouvea, W., Presgrave, O.A., Fingola, F.F., et al. (1991). Neurobehavioral study of the effect of beta-myrcene on rodents. *Braz J Med Biol Res* 24(8), 827-831.

De-Oliveira, A.C., Ribeiro-Pinto, L.F., and Paumgartten, J.R. (1997). In vitro inhibition of CYP2B1 monooxygenase by beta-myrcene and other monoterpenoid compounds. *Toxicol Lett* 92(1), 39-46. doi: 10.1016/s0378-4274(97)00034-9.

Delgado, I.F., Carvalho, R.R., De Almeida Nogueira, A.C.M., Mattos, A.P., Figueiredo, L.H., Oliveira, S.H.P., et al. (1993a). Study on embryo-foetotoxicity of β-myrcene in the rat. *Food and Chemical Toxicology* 31(1), 31-35. doi: https://doi.org/10.1016/0278-6915(93)90175-X.

Delgado, I.F., de Almeida Nogueira, A.C.M., Souza, C.A.M., Costa, A.M.N., Figueiredo, L.H., Mattos, A.P., et al. (1993b). Peri- and postnatal developmental toxicity of β-myrcene in the rat. *Food and Chemical Toxicology* 31(9)**,** 623-628. doi: https://doi.org/10.1016/0278-6915(93)90044-Y.
Doan, T.Q., Ho, D.V., Trong Le, N., Le, A.T., Van Phan, K., Nguyen, H.T., et al. (2019). Chemical composition and anti-inflammatory activity of the essential oil from the leaves of Limnocitrus littoralis (Miq.) Swingle from Vietnam. *Nat Prod Res***,** 1-5. doi: 10.1080/14786419.2019.1652286.
Donati, M., Mondin, A., Chen, Z., Miranda, F.M., do Nascimento, B.B., Jr., Schirato, G., et al. (2015). Radical scavenging and antimicrobial activities of Croton zehntneri, Pterodon emarginatus and Schinopsis brasiliensis essential oils and their major constituents: estragole, trans-anethole, β-caryophyllene and myrcene. *Nat Prod Res* 29(10)**,** 939-946. doi: 10.1080/14786419.2014.964709.
Duarte, I.D., dos Santos, I.R., Lorenzetti, B.B., and Ferreira, S.H. (1992). Analgesia by direct antagonism of nociceptor sensitization involves the arginine-nitric oxide-cGMP pathway. *Eur J Pharmacol* 217(2-3)**,** 225-227. doi: 10.1016/0014-2999(92)90881-4.
Duarte, L.M., Amorim, T.L., Grazul, R.M., and de Oliveira, M.A.L. (2020). Differentiation of aromatic, bittering and dual-purpose commercial hops from their terpenic profiles: An approach involving batch extraction, GC-MS and multivariate analysis. *Food Res Int* 138(Pt A)**,** 109768. doi: 10.1016/j.foodres.2020.109768.
Eri, S., Khoo, B.K., Lech, J., and Hartman, T.G. (2000). Direct Thermal Desorption−Gas Chromatography and Gas Chromatography−Mass Spectrometry Profiling of Hop (Humulus lupulus L.) Essential Oils in Support of Varietal Characterization. *Journal of Agricultural and Food Chemistry* 48(4)**,** 1140-1149. doi: 10.1021/jf9911850.
Fajinmi, O.O., Kulkarni, M.G., Benická, S., Ćavar Zeljković, S., Doležal, K., Tarkowski, P., et al. (2019). Antifungal activity of the volatiles of Agathosma betulina and Coleonema album commercial essential oil and their effect on the morphology of fungal strains Trichophyton rubrum and T. mentagrophytes. *South African Journal of Botany* 122**,** 492-497. doi: https://doi.org/10.1016/j.sajb.2018.03.003.
Ferraz, R.P., Bomfim, D.S., Carvalho, N.C., Soares, M.B., da Silva, T.B., Machado, W.J., et al. (2013). Cytotoxic effect of leaf essential oil of Lippia gracilis Schauer (Verbenaceae). *Phytomedicine* 20(7)**,** 615-621.
Flamini, G., Cosimi, E., Cioni, P.L., Molfetta, I., and Braca, A. (2014). Essential-Oil Composition of Daucus carota ssp. major (Pastinocello Carrot) and Nine Different Commercial Varieties of Daucus carota ssp. sativus Fruits. *Chemistry & Biodiversity* 11(7)**,** 1022-1033. doi: https://doi.org/10.1002/cbdv.201300390.
Foddai, M., Marchetti, M., Ruggero, A., Juliano, C., and Usai, M. (2019). Evaluation of chemical composition and anti-inflammatory, antioxidant, antibacterial activity of essential oil of Sardinian Santolina corsica Jord. & Fourr. *Saudi J Biol Sci* 26(5)**,** 930-937. doi: 10.1016/j.sjbs.2018.08.001.
Forsén, K. (1979). AROMA CONSTITUENTS OF ANGELICA ARCHANGELICA: VARIATIONS IN THE COMPOSITION OF THE ESSENTIAL ROOT OIL OF STRAINS OF VAR. NORVEGICA AND VAR. SATIVA.
Freitas, J.C., Presgrave, O.A., Fingola, F.F., Menezes, M.A., and Paumgartten, F.J. (1993). Effect of beta-myrcene on pentobarbital sleeping time. *Braz J Med Biol Res* 26(5)**,** 519-523.

Friedrich, K., Delgado, I.F., Santos, L.M., and Paumgartten, F.J. (2007). Assessment of sensitization potential of monoterpenes using the rat popliteal lymph node assay. *Food Chem Toxicol* 45(8)**,** 1516-1522. doi: 10.1016/j.fct.2007.02.011.
Gbenou, J.D., Ahounou, J.F., Akakpo, H.B., Laleye, A., Yayi, E., Gbaguidi, F., et al. (2013). Phytochemical composition of Cymbopogon citratus and Eucalyptus citriodora essential oils and their anti-inflammatory and analgesic properties on Wistar rats. *Mol Biol Rep* 40(2)**,** 1127-1134. doi: 10.1007/s11033-012-2155-1.
Gomes-Carneiro, M.R., Viana, M.E.S., Felzenszwalb, I., and Paumgartten, F.J.R. (2005). Evaluation of β-myrcene, α-terpinene and (+)- and (−)-α-pinene in the Salmonella/microsome assay. *Food and Chemical Toxicology* 43(2)**,** 247-252. doi: https://doi.org/10.1016/j.fct.2004.09.011.
Gonçalves, J., Figueira, J., Rodrigues, F., and Câmara, J.S. (2012). Headspace solid-phase microextraction combined with mass spectrometry as a powerful analytical tool for profiling the terpenoid metabolomic pattern of hop-essential oil derived from Saaz variety. *J Sep Sci* 35(17)**,** 2282-2296. doi: 10.1002/jssc.201200244.
Greche, H., Ismaili-Alaoui, M., Zrira, S., Benjilali, B., Belanger, A., and Hajjaji, N. (1999). Composition of Tanacetum annuum L. Oil from Morocco. *Journal of Essential Oil Research* 11(3)**,** 343-348. doi: 10.1080/10412905.1999.9701150.
Guadagni, D.G., Buttery, R.G., and Harris, J. (1966). Odour intensities of hop oil components. *J Sci Food Agric* 17(3)**,** 142-144. doi: 10.1002/jsfa.2740170311.
Gulluni, N., Re, T., Loiacono, I., Lanzo, G., Gori, L., Macchi, C., et al. (2018). Cannabis Essential Oil: A Preliminary Study for the Evaluation of the Brain Effects. *Evidence-Based Complementary and Alternative Medicine* 2018**,** 1709182. doi: 10.1155/2018/1709182.
Gurgel do Vale, T., Couto Furtado, E., Santos, J.G., and Viana, G.S.B. (2002). Central effects of citral, myrcene and limonene, constituents of essential oil chemotypes from Lippia alba (Mill.) N.E. Brown. *Phytomedicine* 9(8)**,** 709-714. doi: https://doi.org/10.1078/094471102321621304.
Hausen, B.M., Reichling, J., and Harkenthal, M. (1999). Degradation products of monoterpenes are the sensitizing agents in tea tree oil. *Am J Contact Dermat* 10(2)**,** 68-77. doi: 10.1016/s1046-199x(99)90002-7.
Höferl, M., Stoilova, I., Schmidt, E., Wanner, J., Jirovetz, L., Trifonova, D., et al. (2014). Chemical Composition and Antioxidant Properties of Juniper Berry (Juniperus communis L.) Essential Oil. Action of the Essential Oil on the Antioxidant Protection of Saccharomyces cerevisiae Model Organism. *Antioxidants (Basel, Switzerland)* 3(1)**,** 81-98. doi: 10.3390/antiox3010081.
Holm, Y., Vuorela, P., and Hiltunen, R. (1997). Enantiomeric composition of monoterpene hydrocarbons in n-hexane extracts of Angelica archangelica L. roots and seeds. *Flavour and Fragrance Journal* 12(6)**,** 397-400. doi: 10.1002/(sici)1099-1026(199711/12)12:6<397::Aid-ffj670>3.0.Co;2-z.
Hoseini, S.M., Yousefi, M., Hoseinifar, S.H., and Van Doan, H. (2019). Antioxidant, enzymatic and hematological responses of common carp (Cyprinus carpio) fed with myrcene- or menthol-supplemented diets and exposed to ambient ammonia. *Aquaculture* 506**,** 246-255. doi: https://doi.org/10.1016/j.aquaculture.2019.03.048.

Huaman, Y., de la Cruz, O.A., Bosilcov, A., and Batiu, I. (2004). Essential oil from the fruits of Schinus molle L. from Peru. *Journal of Essential Oil Bearing Plants* 7(3)**,** 223-227. doi: 10.1080/0972-060X.2004.10643396.

Huang, L., and Capdevila, L. (2017). Aromatherapy Improves Work Performance Through Balancing the Autonomic Nervous System. *J Altern Complement Med* 23(3)**,** 214-221. doi: 10.1089/acm.2016.0061.

Hwang, E., Ngo, H.T.T., Park, B., Seo, S.A., Yang, J.E., and Yi, T.H. (2017). Myrcene, an Aromatic Volatile Compound, Ameliorates Human Skin Extrinsic Aging via Regulation of MMPs Production. *Am J Chin Med* 45(5)**,** 1113-1124. doi: 10.1142/s0192415x17500604.

Jansen, C., Shimoda, L.M.N., Kawakami, J.K., Ang, L., Bacani, A.J., Baker, J.D., et al. (2019). Myrcene and terpene regulation of TRPV1. *Channels* 13(1)**,** 344-366. doi: 10.1080/19336950.2019.1654347.

Jirovetz, L., Buchbauer, G., Fleischhacker, W., and Ngassoum, M.B. (1999). Analysis of leaf volatiles of Zanthoxylum gillettii used in folk medicine of Cameroon. *Planta Med* 65(2)**,** 181-183. doi: 10.1055/s-2006-960463.

Kanode, R., Chandra, S., and Sharma, S. (2017). Application of bacterial reverse mutation assay for detection of non-genotoxic carcinogens. *Toxicol Mech Methods* 27(5)**,** 376-381. doi: 10.1080/15376516.2017.1300616.

Kauderer, B., Zamith, H., Paumgartten, F.J.R., Speit, G., and Holden, H.E. (1991). Evaluation of the mutagenicity of β-myrcene in mammalian cells in vitro. *Environmental and Molecular Mutagenesis* 18(1)**,** 28-34. doi: 10.1002/em.2850180106.

Kim, J.-O., Y.-S. Kim, K.-H. Lee, M.-N. Kim, S.-H. Rhee, S.-H. Moon, and K.-Y. Park (1992). Antimutagenic effect of the major volatile compounds identified from mugwort (Artemisia asictica nakai) leaves. *J. Korean Soc. Food Nutr.* 21(3)**,** 308-313.

Kjeldsen, F., Christensen, L.P., and Edelenbos, M. (2003). Changes in Volatile Compounds of Carrots (Daucus carota L.) During Refrigerated and Frozen Storage. *Journal of Agricultural and Food Chemistry* 51(18)**,** 5400-5407. doi: 10.1021/jf030212q.

Kligman, A. (1972). Report to RIFM, 25 August.

Kurobayashi, Y., Kouno, E., Fujita, A., Morimitsu, Y., and Kubota, K. (2006). Potent Odorants Characterize the Aroma Quality of Leaves and Stalks in Raw and Boiled Celery. *Bioscience, Biotechnology, and Biochemistry* 70(4)**,** 958-965. doi: 10.1271/bbb.70.958.

Lee, J.-H., Lee, K., Lee, D.H., Shin, S.Y., Yong, Y., and Lee, Y.H. (2015). Anti-invasive effect of β-myrcene, a component of the essential oil from Pinus koraiensis cones, in metastatic MDA-MB-231 human breast cancer cells. *Journal of the Korean Society for Applied Biological Chemistry* 58(4)**,** 563-569. doi: 10.1007/s13765-015-0081-3.

Lim, S.S., Shin, K.H., Ban, H.S., Kim, Y.P., Jung, S.H., Kim, Y.J., et al. (2002). Effect of the essential oil from the flowers of Magnolia sieboldii on the lipopolysaccharide-induced production of nitric oxide and prostaglandin E2 by rat peritoneal macrophages. *Planta Med* 68(5)**,** 459-462. doi: 10.1055/s-2002-32085.

Liu, Q., Li, D., Wang, W., Wang, D., Meng, X., and Wang, Y. (2016). Chemical Composition and Antioxidant Activity of Essential Oils and Methanol Extracts of Different Parts from Juniperus rigida Siebold & Zucc. *Chemistry & Biodiversity* 13(9)**,** 1240-1250. doi: 10.1002/cbdv.201600048.

Liu, S.-M., Wang, S.-J., Song, S.-Y., Zou, Y., Wang, J.-R., and Sun, B.-Y. (2017). Characteristic differences in essential oil composition of six Zanthoxylum bungeanum Maxim. (Rutaceae) cultivars and their biological significance. *Journal of Zhejiang University. Science. B* 18(10)**,** 917-920. doi: 10.1631/jzus.B1700232.

Lorenzetti, B.B., Souza, G.E., Sarti, S.J., Santos Filho, D., and Ferreira, S.H. (1991). Myrcene mimics the peripheral analgesic activity of lemongrass tea. *J Ethnopharmacol* 34(1)**,** 43-48. doi: 10.1016/0378-8741(91)90187-i.

Lu, H., Wu, X., Liang, Y., and Zhang, J. (2006). Variation in chemical composition and antibacterial activities of essential oils from two species of Houttuynia THUNB. *Chem Pharm Bull (Tokyo)* 54(7)**,** 936-940. doi: 10.1248/cpb.54.936.

Maggi, F., Fortuné Randriana, R., Rasoanaivo, P., Nicoletti, M., Quassinti, L., Bramucci, M., et al. (2013). Chemical composition and in vitro biological activities of the essential oil of Vepris macrophylla (BAKER) I.VERD. endemic to Madagascar. *Chem Biodivers* 10(3)**,** 356-366. doi: 10.1002/cbdv.201200253.

Malizia, R.A., Molli, J.S., Cardell, D.A., and Grau, R.J. (1999). Essential oil of hop cones (Humulus lupulus L.). *Journal of Essential Oil Research* 11(1)**,** 13-15.

Malti, C.E.W., Baccati, C., Mariani, M., Hassani, F., Babali, B., Atik-Bekkara, F., et al. (2019). Biological Activities and Chemical Composition of Santolina africana Jord. et Fourr. Aerial Part Essential Oil from Algeria: Occurrence of Polyacetylene Derivatives. *Molecules* 24(1). doi: 10.3390/molecules24010204.

Matura, M., Sköld, M., Börje, A., Andersen, K.E., Bruze, M., Frosch, P., et al. (2005). Selected oxidized fragrance terpenes are common contact allergens. *Contact Dermatitis* 52(6)**,** 320-328. doi: 10.1111/j.0105-1873.2005.00605.x.

MEINER, C., and MEDIAVILLA, V. (1998). Factors influencing the yield and the quality of hemp essentials oil. *Journal of the International Hemp Association* 5**,** 16-20.

Mikyška, A., and Olšovská, J. "Czech research and development in the field of brewing raw materials".).

Mirghaed, A.T., Yasari, M., Mirzargar, S.S., and Hoseini, S.M. (2018). Rainbow trout (Oncorhynchus mykiss) anesthesia with myrcene: efficacy and physiological responses in comparison with eugenol. *Fish Physiol Biochem* 44(3)**,** 919-926. doi: 10.1007/s10695-018-0481-5.

Mitić-Ćulafić, D., Žegura, B., Nikolić, B., Vuković-Gačić, B., Knežević-Vukčević, J., and Filipič, M. (2009). Protective effect of linalool, myrcene and eucalyptol against t-butyl hydroperoxide induced genotoxicity in bacteria and cultured human cells. *Food and Chemical Toxicology* 47(1)**,** 260-266. doi: https://doi.org/10.1016/j.fct.2008.11.015.

Moreno, O.M. (1972). Acute oral toxicity studies of myrcene in mice, rats and rabbits. *Unpublished report to the Research Institute of Fragrance Materials*.

Munafo, J.P., Didzbalis, J., Schnell, R.J., and Steinhaus, M. (2016). Insights into the Key Aroma Compounds in Mango (Mangifera indica L. 'Haden') Fruits by Stable Isotope Dilution Quantitation and Aroma Simulation Experiments. *Journal of Agricultural and Food Chemistry* 64(21)**,** 4312-4318. doi: 10.1021/acs.jafc.6b00822.

Nance, M., and Setzer, W. (2011). Volatile components of aroma hops (Humulus lupulus L.) commonly used in beer brewing. *Journal of Brewing and Distilling* 2**,** 16-22.

Newmark, F.M. (1978). Hops allergy and terpene sensitivity: an occupational disease. *Ann Allergy* 41(5)**,** 311-312.
Nivinskiene, O., Butkiene, R., and Mockute, D. (2007). The Seed (Fruit) Essential Oils of Angelica archangelica L. Growing Wild in Lithuania. *Journal of Essential Oil Research* 19(5)**,** 477-481. doi: 10.1080/10412905.2007.9699957.
Nogueira de Melo, G.A., Grespan, R., Fonseca, J.P., Farinha, T.O., da Silva, E.L., Romero, A.L., et al. (2011a). Inhibitory effects of ginger (Zingiber officinale Roscoe) essential oil on leukocyte migration in vivo and in vitro. *J Nat Med* 65(1)**,** 241-246. doi: 10.1007/s11418-010-0479-5.
Nogueira de Melo, G.A., Grespan, R., Fonseca, J.P., Farinha, T.O., Silva, E.L., Romero, A.L., et al. (2011b). Rosmarinus officinalis L. essential oil inhibits in vivo and in vitro leukocyte migration. *J Med Food* 14(9)**,** 944-946. doi: 10.1089/jmf.2010.0159.
NTP (2010). "NTP technical report on the toxicology and carcinogenesis studies of β-myrcene (CAS No. 123-35-3) in F344/N rats and B6C3F1 mice (gavage studies). Research Triangle Park, NC, National Institutes of Health. 2010".).
Ohtsubo, S., Fujita, T., Matsushita, A., and Kumamoto, E. (2015). Inhibition of the compound action potentials of frog sciatic nerves by aroma oil compounds having various chemical structures. *Pharmacol Res Perspect* 3(2)**,** e00127. doi: 10.1002/prp2.127.
Ojala, A., Huopalahti, R., NykÄNen, A., and Kallio, H. (1986). Variation of Angelica archangelica subsp. archangelica (Apiaceae) in northern Fennoscandia: 5. Variation in composition of essential oil. *Annales Botanici Fennici* 23(4)**,** 325-332.
Okamura, K., Iwakami, S., and Matsunaga, T. (1993). Biological activity of monoterpenes from trees. *Toyama-Ken Yakuji Kenkyusho Nenpo* 20**,** 95-101.
Oliveira, D.R., Leitão, G.G., Santos, S.S., Bizzo, H.R., Lopes, D., Alviano, C.S., et al. (2006). Ethnopharmacological study of two Lippia species from Oriximiná, Brazil. *Journal of Ethnopharmacology* 108(1)**,** 103-108. doi: https://doi.org/10.1016/j.jep.2006.04.018.
Orlando, J.B., Silva, B.O., Pires-Cunha, C.L., Hiruma-Lima, C.A., Gaivão, I.O.N.d.M., and Maistro, E.L. (2019). Genotoxic effects induced by beta-myrcene following metabolism by liver HepG2/C3A human cells. *Journal of Toxicology and Environmental Health, Part A* 82(3)**,** 176-185. doi: 10.1080/15287394.2019.1577195.
Paaver, U., Orav, A., Arak, E., Mäeorg, U., and Raal, A. (2008). Phytochemical analysis of the essential oil of Thymus serpyllum L. growing wild in Estonia. *Natural Product Research* 22(2)**,** 108-115. doi: 10.1080/14786410601035118.
Palá-Paúl, J., Pérez-Alonso, M.J., Velasco-Negueruela, A., Palá-Paúl, R., Sanz, J., and Conejero, F. (2001). Seasonal variation in chemical constituents of Santolina rosmarinifolia L. ssp. rosmarinifolia. *Biochemical Systematics and Ecology* 29(7)**,** 663-672. doi: https://doi.org/10.1016/S0305-1978(01)00032-1.
Paraschos, S., Magiatis, P., Gikas, E., Smyrnioudis, I., and Skaltsounis, A.-L. (2016). Quality profile determination of Chios mastic gum essential oil and detection of adulteration in mastic oil products with the application of chiral and non-chiral GC–MS analysis. *Fitoterapia* 114**,** 12-17. doi: https://doi.org/10.1016/j.fitote.2016.08.003.
Park, C., Juliani, H., Park, H., Yu, H., and Simon, J. (2003). Comparison of essential oil composition between Angelica gigas and Angelica acutiloba. *Plant Resources* 6(3)**,** 183-187.

Pasqua, G., Monacelli, B., Silvestrini, A., and Manganaro, R. (2001). In vitro root differentiation and essential-oil accumulation in Angelica archangelica. *In Vitro Cellular & Developmental Biology - Plant* 37(6)**,** 763-766. doi: 10.1007/s11627-001-0126-7.

Paula-Freire, L.I., Andersen, M.L., Molska, G.R., Köhn, D.O., and Carlini, E.L. (2013). Evaluation of the antinociceptive activity of Ocimum gratissimum L. (Lamiaceae) essential oil and its isolated active principles in mice. *Phytother Res* 27(8)**,** 1220-1224. doi: 10.1002/ptr.4845.

Paula-Freire, L.I., Molska, G.R., Andersen, M.L., and Carlini, E.L. (2016). Ocimum gratissimum Essential Oil and Its Isolated Compounds (Eugenol and Myrcene) Reduce Neuropathic Pain in Mice. *Planta Med* 82(3)**,** 211-216. doi: 10.1055/s-0035-1558165.

Paumgartten, F.J., De-Carvalho, R.R., Souza, C.A., Madi, K., and Chahoud, I. (1998). Study of the effects of beta-myrcene on rat fertility and general reproductive performance. *Braz J Med Biol Res* 31(7)**,** 955-965. doi: 10.1590/s0100-879x1998000700012.

Paumgartten, F.J., Delgado, I.F., Alves, E.N., Nogueira, A.C., de-Farias, R.C., and Neubert, D. (1990). Single dose toxicity study of beta-myrcene, a natural analgesic substance. *Braz J Med Biol Res* 23(9)**,** 873-877.

Paventi, G., de Acutis, L., De Cristofaro, A., Pistillo, M., Germinara, G.S., and Rotundo, G. (2020). Biological Activity of Humulus Lupulus (L.) Essential Oil and Its Main Components Against Sitophilus granarius (L.). *Biomolecules* 10(8)**,** 1108. doi: 10.3390/biom10081108.

Petropoulos, S., Daferera, D., Akoumianakis, C., Passam, H., and Polissiou, M. (2004). The effect of sowing date and growth stage on the essential oil composition of three types of parsley (Petroselinum crispum). *Journal of the Science of Food and Agriculture* 84(12)**,** 1606-1610. doi: https://doi.org/10.1002/jsfa.1846.

Philippe, S., Farougou, S., Azokpota, P., Youssao Abdou Karim, I., Fanou, B., Kaneho, S., et al. (2012). IN VITRO ANTIFUNGAL ACTIVITY OF ESSENTIAL OIL OF PIMENTA RACEMOSA AGAINST FUNGAL ISOLATES FROM WAGASHI, A TRADITIONAL CHEESE PRODUCED IN BENIN. *International Journal of Natural and Applied Sciences ISSN: 0794 – 4713* 8**,** 25-34.

Pinto, Z.T., Sánchez, F.F., dos Santos, A.R., Amaral, A.C., Ferreira, J.L., Escalona-Arranz, J.C., et al. (2015). Chemical composition and insecticidal activity of Cymbopogon citratus essential oil from Cuba and Brazil against housefly. *Rev Bras Parasitol Vet* 24(1)**,** 36-44. doi: 10.1590/s1984-29612015006.

Rao, V.S., Menezes, A.M., and Viana, G.S. (1990). Effect of myrcene on nociception in mice. *J Pharm Pharmacol* 42(12)**,** 877-878. doi: 10.1111/j.2042-7158.1990.tb07046.x.

Rasooli, I., and Mirmostafa, S.A. (2003). Bacterial susceptibility to and chemical composition of essential oils from Thymus kotschyanus and Thymus persicus. *J Agric Food Chem* 51(8)**,** 2200-2205. doi: 10.1021/jf0261755.

Raut, S., von Gersdorff, G.J., Münsterer, J., Kammhuber, K., Hensel, O., and Sturm, B. Influence of pre-drying storage time on essential oil components in dried hops (Humulus lupulus L.). *Journal of the Science of Food and Agriculture* n/a(n/a). doi: https://doi.org/10.1002/jsfa.10844.

Röscheisen, C., Zamith, H., Paumgartten, F.J., and Speit, G. (1991). Influence of β-myrcene on sister-chromatid exchanges induced by mutagens in V79 and HTC cells. *Mutation Research Letters* 264(1)**,** 43-49.

Roslon, W., Wajs-Bonikowska, A., Geszprych, A., and Osinska, E. (2016). Characteristics of Essential Oil from Young Shoots of Garden Angelica (Angelica archangelica L.). *Journal of Essential Oil Bearing Plants* 19(6)**,** 1462-1470. doi: 10.1080/0972060X.2016.1238322.

Rudloff, E.V. (1985). The leaf oil terpene composition of eastern white pine, Pinus strobus L. *Flavour and Fragrance Journal* 1(1)**,** 33-35. doi: https://doi.org/10.1002/ffj.2730010108.

Rufino, A.T., Ribeiro, M., Sousa, C., Judas, F., Salgueiro, L., Cavaleiro, C., et al. (2015). Evaluation of the anti-inflammatory, anti-catabolic and pro-anabolic effects of E-caryophyllene, myrcene and limonene in a cell model of osteoarthritis. *Eur J Pharmacol* 750**,** 141-150. doi: 10.1016/j.ejphar.2015.01.018.

Ruiz del Castillo, M.L., and Dobson, G. (2002). Varietal differences in terpene composition of blackcurrant (Ribes nigrum L) berries by solid phase microextraction/gas chromatography. *Journal of the Science of Food and Agriculture* 82(13)**,** 1510-1515. doi: 10.1002/jsfa.1210.

Russin, W.A., Hoesly, J.D., Elson, C.E., Tanner, M.A., and Gould, M.N. (1989). Inhibition of rat mammary carcinogenesis by monoterpenoids. *Carcinogenesis* 10(11)**,** 2161-2164. doi: 10.1093/carcin/10.11.2161.

Sáez, F. (2001). Volatile oil variability in Thymus serpylloides ssp. gadorensis growing wild in Southeastern Spain. *Biochemical Systematics and Ecology* 29(2)**,** 189-198. doi: https://doi.org/10.1016/S0305-1978(00)00040-5.

Saleh, M.M.H., F.A.; Glombitza, K.W. (1998). Cytotoxicity and in vitro effects on human cancer cell lines of volatiles of Apium graveolens var. filicum. *Pharmaceutical & Pharmacological Letters* 8(2)**,** 97-99.

Sawamura, M., Sun, S.H., Ozaki, K., Ishikawa, J., and Ukeda, H. (1999). Inhibitory Effects of Citrus Essential Oils and Their Components on the Formation of N-Nitrosodimethylamine. *Journal of Agricultural and Food Chemistry* 47(12)**,** 4868-4872. doi: 10.1021/jf9903206.

Schmidt, C., and Biendl, M. (2016). Headspace Trap GC-MS analysis of hop aroma compounds in beer. *BrewingScience* 69**,** 9-15.

Shah, S.M., Ullah, F., Shah, S.M., Zahoor, M., and Sadiq, A. (2012). Analysis of chemical constituents and antinociceptive potential of essential oil of Teucrium Stocksianum bioss collected from the North West of Pakistan. *BMC Complement Altern Med* 12**,** 244. doi: 10.1186/1472-6882-12-244.

Shao, Q., Liu, H., Zhang, A., Wan, Y., Hu, R., and Li, M. (2014). Analysis of volatile components extracted from the peels of four different Chinese pomelos using TDS-GC-MS. *Journal of the Science of Food and Agriculture* 94(15)**,** 3248-3254. doi: https://doi.org/10.1002/jsfa.6677.

Silva, S.L.d., Figueiredo, P.M., and Yano, T. (2007). Cytotoxic evaluation of essential oil from Zanthoxylum rhoifolium Lam. leaves. *Acta Amazonica* 37**,** 281-286.

Sousa, O.V., Silvério, M.S., Del-Vechio-Vieira, G., Matheus, F.C., Yamamoto, C.H., and Alves, M.S. (2008). Antinociceptive and anti-inflammatory effects of the essential oil from Eremanthus erythropappus leaves. *J Pharm Pharmacol* 60(6)**,** 771-777. doi: 10.1211/jpp.60.6.0013.

Southwell, I., Russell, M., Smith, R., Brophy, J., and Day, J. (Year). "Melaleuca teretifolia, a novel aromatic and medicinal plant from Australia", in: *III WOCMAP Congress on Medicinal and Aromatic Plants-Volume 3: Perspectives in Natural Product Chemistry 677*), 79-83.

Souza, M., Siani, A.C., Ramos, M., Menezes-de-Lima, O., and Henriques, M. (2003). Evaluation of anti-inflammatory activity of essential oils from two Asteraceae species. *Die Pharmazie* 58**,** 582-586.

Svoboda, K., Jb, H., and Hall, L. (Year). "Investigation of Frankincense producing Boswellia ssp. from Somalia and quest for quality standards").

Tabanca, N., Gao, Z., Demirci, B., Techen, N., Wedge, D.E., Ali, A., et al. (2014). Molecular and phytochemical investigation of Angelica dahurica and Angelica pubescentis essential oils and their biological activity against Aedes aegypti, Stephanitis pyrioides, and Colletotrichum species. *J Agric Food Chem* 62(35)**,** 8848-8857. doi: 10.1021/jf5024752.

Tabanca, N., Nalbantsoy, A., Kendra, P.E., Demirci, F., and Demirci, B. (2020). Chemical Characterization and Biological Activity of the Mastic Gum Essential Oils of Pistacia lentiscus Var. Chia from Turkey. *Molecules* 25(9). doi: 10.3390/molecules25092136.

Tanoh, E.A., Boué, G.B., Nea, F., Genva, M., Wognin, E.L., Ledoux, A., et al. (2020). Seasonal Effect on the Chemical Composition, Insecticidal Properties and Other Biological Activities of Zanthoxylum leprieurii Guill. & Perr. Essential oils. *Foods (Basel, Switzerland)* 9(5)**,** 550. doi: 10.3390/foods9050550.

Tavares, A.C., Gonçalves, M.J., Cruz, M.T., Cavaleiro, C., Lopes, M.C., Canhoto, J., et al. (2010). Essential oils from Distichoselinum tenuifolium: chemical composition, cytotoxicity, antifungal and anti-inflammatory properties. *J Ethnopharmacol* 130(3)**,** 593-598. doi: 10.1016/j.jep.2010.05.054.

Tian, J., Zhang, R., Weng, Y., Qin, Q., Zhang, X., Liu, A., et al. (2020). Myrcene enhances the cardioprotective effect through matrix remodelling in an experimental model of heart failure. *Archives of Medical Science*. doi: 10.5114/aoms.2020.95875.

Trung, H.D., Thang, T.D., Ban, P.H., Hoi, T.M., Dai, D.N., and Ogunwande, I.A. (2014). Terpene constituents of the leaves of five Vietnamese species of Clausena (Rutaceae). *Natural Product Research* 28(9)**,** 622-630. doi: 10.1080/14786419.2014.888555.

Tucker, A.O., Maciarello, M.J., Adams, R.P., Landrum, L.R., and Zanoni, T.A. (1991). Volatile Leaf Oils of Caribbean Myrtaceae. I. Three Varieties of Pimenta racemosa (Miller) J. Moore of the Dominican Republic and the Commercial Bay Oil. *Journal of Essential Oil Research* 3(5)**,** 323-329. doi: 10.1080/10412905.1991.9697952.

Tyśkiewicz, K., Gieysztor, R., Konkol, M., Szałas, J., and Rój, E. (2018). Essential Oils from Humulus Lupulus scCO_2 Extract by Hydrodistillation and Microwave-Assisted Hydrodistillation. *Molecules (Basel, Switzerland)* 23(11)**,** 2866. doi: 10.3390/molecules23112866.

Umezu, T., Ito, H., Nagano, K., Yamakoshi, M., Oouchi, H., Sakaniwa, M., et al. (2002). Anticonflict effects of rose oil and identification of its active constituents. *Life Sci* 72(1)**,** 91-102. doi: 10.1016/s0024-3205(02)02197-5.

Umezu, T., Nagano, K., Ito, H., Kosakai, K., Sakaniwa, M., and Morita, M. (2006). Anticonflict effects of lavender oil and identification of its active constituents. *Pharmacol Biochem Behav* 85(4)**,** 713-721. doi: 10.1016/j.pbb.2006.10.026.

Uyeda, S., Sharmin, T., Satho, T., Irie, K., Watanabe, M., Hosokawa, M., et al. (2016). Enhancement and regulation effect of myrcene on antibody response in immunization with ovalbumin and Ag85B in mice. *Asian Pac J Allergy Immunol* 34(4)**,** 314-323. doi: 10.12932/ap0734.

Van Opstaele, F., De Causmaecker, B., Aerts, G., and De Cooman, L. (2012). Characterization of novel varietal floral hop aromas by headspace solid phase microextraction and gas chromatography-mass spectrometry/olfactometry. *J Agric Food Chem* 60(50)**,** 12270-12281. doi: 10.1021/jf304421d.

Vernin, G. (1991). Volatile Constituents of the Essential Oil of Santolina chamaecyparissus L. *Journal of Essential Oil Research* 3(1)**,** 49-53. doi: 10.1080/10412905.1991.9697907.

Viana, G.S., do Vale, T.G., Silva, C.M., and Matos, F.J. (2000). Anticonvulsant activity of essential oils and active principles from chemotypes of Lippia alba (Mill.) N.E. Brown. *Biol Pharm Bull* 23(11)**,** 1314-1317. doi: 10.1248/bpb.23.1314.

Vinitketkumnuen, U., Puatanachokchai, R., Kongtawelert, P., Lertprasertsuke, N., and Matsushima, T. (1994). Antimutagenicity of lemon grass (Cymbopogon citratus Stapf) to various known mutagens in salmonella mutation assay. *Mutat Res* 341(1)**,** 71-75. doi: 10.1016/0165-1218(94)90025-6.

Wahab, I.R., Blagojević, P.D., Radulović, N.S., and Boylan, F. (2011). Volatiles of Curcuma mangga Val. & Zijp (Zingiberaceae) from Malaysia. *Chem Biodivers* 8(11)**,** 2005-2014. doi: 10.1002/cbdv.201100135.

Yu, Z., Wang, B., Yang, F., Sun, Q., Yang, Z., and Zhu, L. (2011). Chemical Compositionand Anti-acetyl cholinesterase Activity of Flower Essential Oils of Artemisiaannuaat Different Flowering Stage. *Iranian journal of pharmaceutical research : IJPR* 10(2)**,** 265-271.

ZAHED, N., HOSNI, K., BRAHIM, N.B., and SEBEI, H. (2011). ESSENTIAL OIL COMPOSITION OF SCHINUS MOLLE L. FRUITS: AN ORNAMENTAL SPECIES USED AS CONDIMENT. *Journal of Food Biochemistry* 35(2)**,** 400-408. doi: https://doi.org/10.1111/j.1745-4514.2010.00391.x.

Zaiter, L., Benayache, F., Beghidja, N., Figueredo, G., Chalard, P., Chalchat, J.-C., et al. (2015). Essential oils of Santolina africana Jord. & Fourr. and Santolina chamaecyparissus L. *Journal of Essential Oil Bearing Plants* 18(6)**,** 1338-1342. doi: 10.1080/0972060X.2014.884769.

Zamith, H.P., Vidal, M.N., Speit, G., and Paumgartten, F.J. (1993). Absence of genotoxic activity of beta-myrcene in the in vivo cytogenetic bone marrow assay. *Braz J Med Biol Res* 26(1)**,** 93-98.

Zeller, A., and Rychlik, M. (2006). Character Impact Odorants of Fennel Fruits and Fennel Tea. *Journal of Agricultural and Food Chemistry* 54(10)**,** 3686-3692. doi: 10.1021/jf052944j.
